# GWAS of Leaf Morphological and Chlorophyll-Related Traits Provides Genetic Resources for Improving Photosynthetic Traits in Sugarcane

**DOI:** 10.3390/plants15193000

**Published:** 2026-09-30

**Authors:** Yongsheng Chen, Huiyi He, Xinglong Chen, Jiayun Wu, Nannan Zhang, Jianwu Wang

**Affiliations:** 1 College of Natural Resources and Environment, South China Agricultural University, Guangzhou 510642, China; yongshengchen100@126.com; 2 Guangdong Sugarcane Genetic Improvement Engineering Center, Institute of Nanfan & Seed Industry, Guangdong Academy of Science, Guangzhou 510316, China; emil06@126.com (H.H.); chenxinglong567@126.com (X.C.); jiayunng@163.com (J.W.)

**Keywords:** *Saccharum* spp., leaf morphology traits, chlorophyll-related traits, SNPs, candidate genes, GWAS

## Abstract

Photosynthesis is the ultimate source of energy for crop productivity, and leaf morphology as well as chlorophyll content are traditional, indirectly related traits of photosynthesis. However, the genetic basis of these traits remains unexplored in sugarcane. In this study, we conducted genome-wide association studies (GWAS) to identify loci controlling nine photosynthesis-related traits, including four leaf morphological traits (leaf length, leaf width, leaf ratio, and leaf area) and five chlorophyll-related traits (chlorophyll a, chlorophyll b, chlorophyll a/b ratio, total chlorophyll, and SPAD value). The analysis was performed using whole-genome resequencing data from 219 elite, globally diverse sugarcane materials evaluated at two locations across two years (four environments). Based on 5,964,084 high-quality single nucleotide polymorphisms (SNPs), we identified 308 GWAS-significant SNPs (*p* ≤ 1.5 × 10^−6^) that were consistently detected in two or more environments, defining 97 GWAS-associated intervals across 30 chromosomes. Within these intervals, 98 putative genes were predicted, of which 20 genes in 16 intervals were potentially associated with leaf photosynthesis based on functional annotation and literature mining. These candidates are primarily involved in pathways related to ADP-ribosylation factors, ATP-dependent Clp protease proteolytic subunits, protein kinases, F-box proteins, MYB and WRKY transcription factors, UVR8, pentatricopeptide repeat-containing (PPR) proteins, Aux/IAA proteins, monogalactosyldiacylglycerol (MGDG) synthase, Orange (OR) proteins, PsbP domain-containing (PPD) proteins, and chloroplast stem-loop binding proteins of 41 kDa b (CSP41b). Ten genes were prioritized for future functional validation. Notably, these photosynthesis-related structural and pigment traits represent a different regulatory layer from direct gas exchange parameters (e.g., net photosynthetic rate), and their associations with actual photosynthetic efficiency require further validation. Nevertheless, this study provides novel candidate genes and molecular markers to assist marker-assisted breeding aimed at improving the photosynthetic structure and light-harvesting capacity of sugarcane.

## 1. Introduction

Amid the escalating global food security crisis, improving crop yields remains a central objective of agricultural research [1]. Photosynthesis underpins all life on Earth and 30represents the ultimate biological source of energy. Identifying key genes that regulate photosynthesis through molecular genetic approaches is therefore a critical pathway to improving photosynthetic efficiency [2].

Chlorophyll is the fundamental pigment enabling photosynthesis in plants [3]. Accordingly, leaf chlorophyll content, often measured via SPAD values, is a widely used proxy for photosynthetic capacity and has been extensively investigated across diverse crop species [4]. Leaf size is another classical and critical trait that influences light interception and photosynthetic efficiency, making it a long-standing target for yield improvement [5]. Positive correlations between increased leaf size and yield have been reported in rice [6]. In particular, the morphology and functionality of photosynthetically active leaves directly affect photosynthetic efficiency and grain yield in rice. Thus, elucidating the genetic basis of leaf size and shape is of substantial importance for optimizing leaf architecture, photosynthetic performance, and yield [7].

In earlier studies, many significant single nucleotide polymorphisms (SNPs) and candidate genes were identified using genome-wide association studies (GWAS) for leaf chlorophyll content and leaf morphology traits. For instance, researchers have reported that GWAS on leaf chlorophyll content, measured via SPAD values, was performed in peanut [3], maize [8], and rice [4]. In wheat, two independent GWAS approaches identified a novel quantitative trait locus (QTL) for the flag leaf ratio [9]. In rice, 91 QTLs associated with four leaf shape traits were detected by GWAS, among which five candidate genes were identified [7]. It was also reported that GWAS identified 26 significant SNPs associated with leaf morphology in alfalfa [5]. Despite these advances, previous GWAS efforts on chlorophyll content, SPAD values, and leaf morphology have only partially captured the genetic basis of photosynthesis-related traits. Photosynthesis is an inherently complex system encompassing numerous biophysical and biochemical processes, including light absorption, energy transduction, and carbon metabolism [10]. As a result, many identified QTLs remain difficult to validate or fully exploit in breeding programs [1,11].

Sugarcane (*Saccharum* spp.), a C_4_ grass, is the world’s most important crop for sugar and bioenergy production [12] and is characterized by exceptionally high photosynthetic efficiency [13]. GWAS has been widely applied in sugarcane to identify loci associated with cane yield [14,15], sucrose content [14,15,16,17,18], agronomic traits [14,15,17,19], fiber content [18,20,21], disease resistance [22], and leaf angle [23]. In contrast, the genetic basis of photosynthesis-related traits in sugarcane has received comparatively little attention.

On the other hand, sugarcane possesses one of the most complex crop genomes, with modern cultivars being interspecific hybrids between *S. officinarum* (x = 10, 2n ≈ 80) and *S. spontaneum* (x = 8, 2n ≈ 64–128), resulting in highly polyploid and aneuploid chromosome compositions. This complexity poses substantial challenges for trait dissection via GWAS, as standard methods assume diploid biallelic SNPs and cannot fully capture allele dosage effects. Nevertheless, given the availability of a high-quality reference genome (AP85-441) and large-scale resequencing, GWAS remains a powerful hypothesis-generating approach for this crop.

In this study, we aimed to: (i) evaluate the phenotypic variation and heritability of nine leaf morphology and chlorophyll traits across four environments in a diverse panel of 219 sugarcane materials; (ii) identify intervals associated with these traits via GWAS; (iii) predict candidate genes within GWAS-associated intervals and cross-reference them with known photosynthetic pathways; and (iv) provide genetic resources for breeding sugarcane with improved leaf photosynthetic traits. Given that genotype × environment (G×E) interactions can substantially influence leaf development and photosynthetic performance, we further assessed the consistency of genetic effects across environments to identify stable associations.

Leveraging our previous whole-genome resequencing of 219 elite sugarcane materials and the identification of approximately six million high-quality SNPs [14], we performed a complementary GWAS to systematically dissect the genetic basis of leaf chlorophyll content and leaf size, thereby extending our earlier analysis of leaf photosynthetic gas exchange parameters [24]. It should be noted that leaf morphology and chlorophyll-related traits are indirect proxies of photosynthesis and do not directly measure photosynthetic efficiency, as they are distinct from gas exchange parameters such as net photosynthetic rate, stomatal conductance, and transpiration. To our knowledge, this is the first GWAS targeting the basic traits of sugarcane photosynthetic structure, namely leaf morphology and chlorophyll content. This study fills a gap in the genetic research of sugarcane photosynthesis by examining the association between light-harvesting structure and photosynthetic output.

## 2. Results

### 2.1. Phenotypic Variation, Distribution, and Multi-Environment Comparison of Nine Photosynthesis-Related Traits

**Figure 1:**
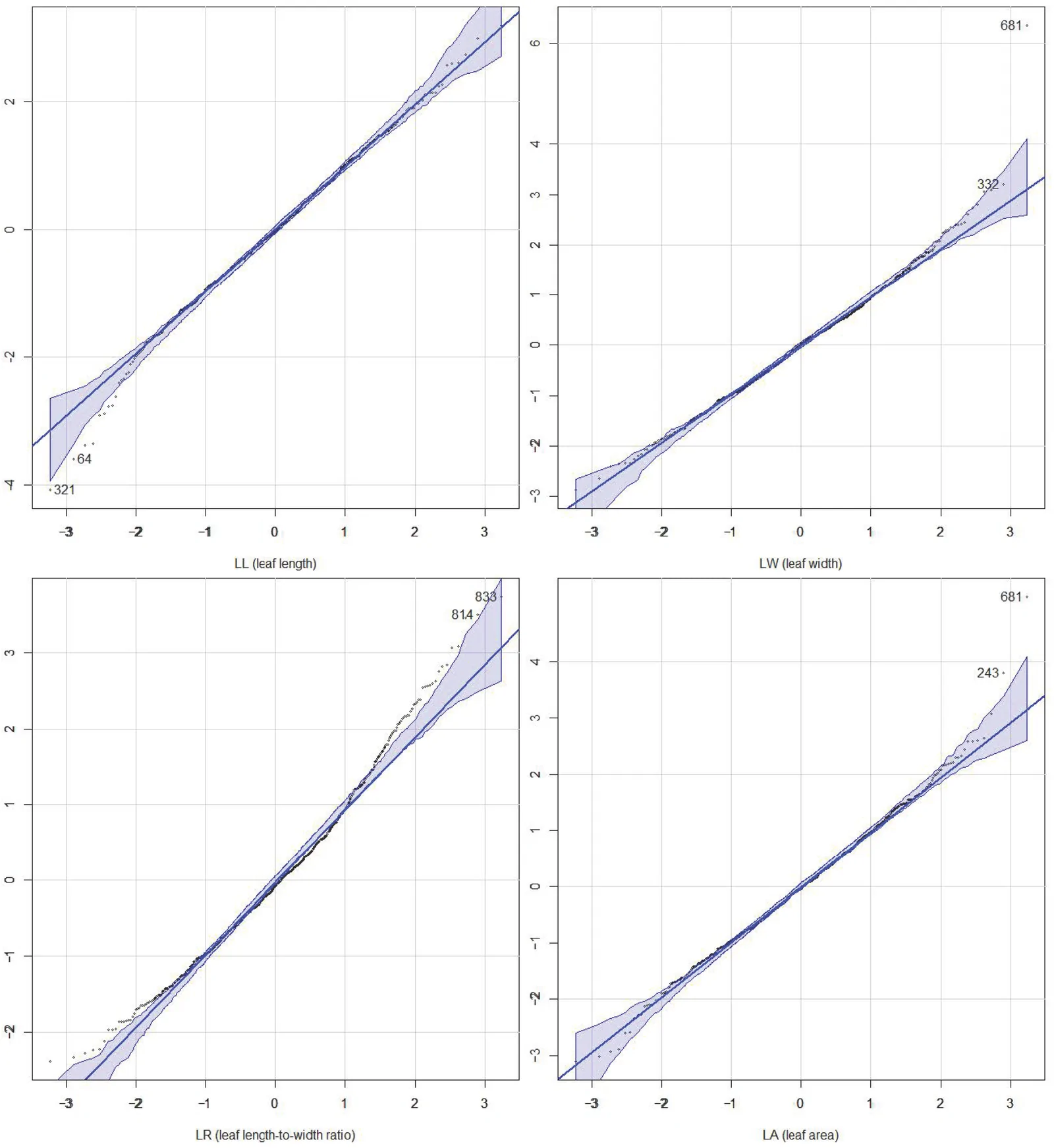
A quantile–quantile (Q-Q) plot of normal distribution for the phenotypic data of the four leaf morphology traits across four environments. The four environments were H1 (Wengyuan, June–July 2023), H2 (Wengyuan, September 2023), H3 (Zhanjiang, September 2023), and H4 (Wengyuan, July–August 2022). The panels show (left to right, top to bottom): leaf length (LL, cm), leaf width (LW, cm), leaf ratio LW/LL (LR, dimensionless), and leaf area (LA, cm^2^). The blue lines represented the theoretical quantile line (*y* = *x*), and the blue shaded areas indicated the 95% confidence interval.

**Figure 2:**
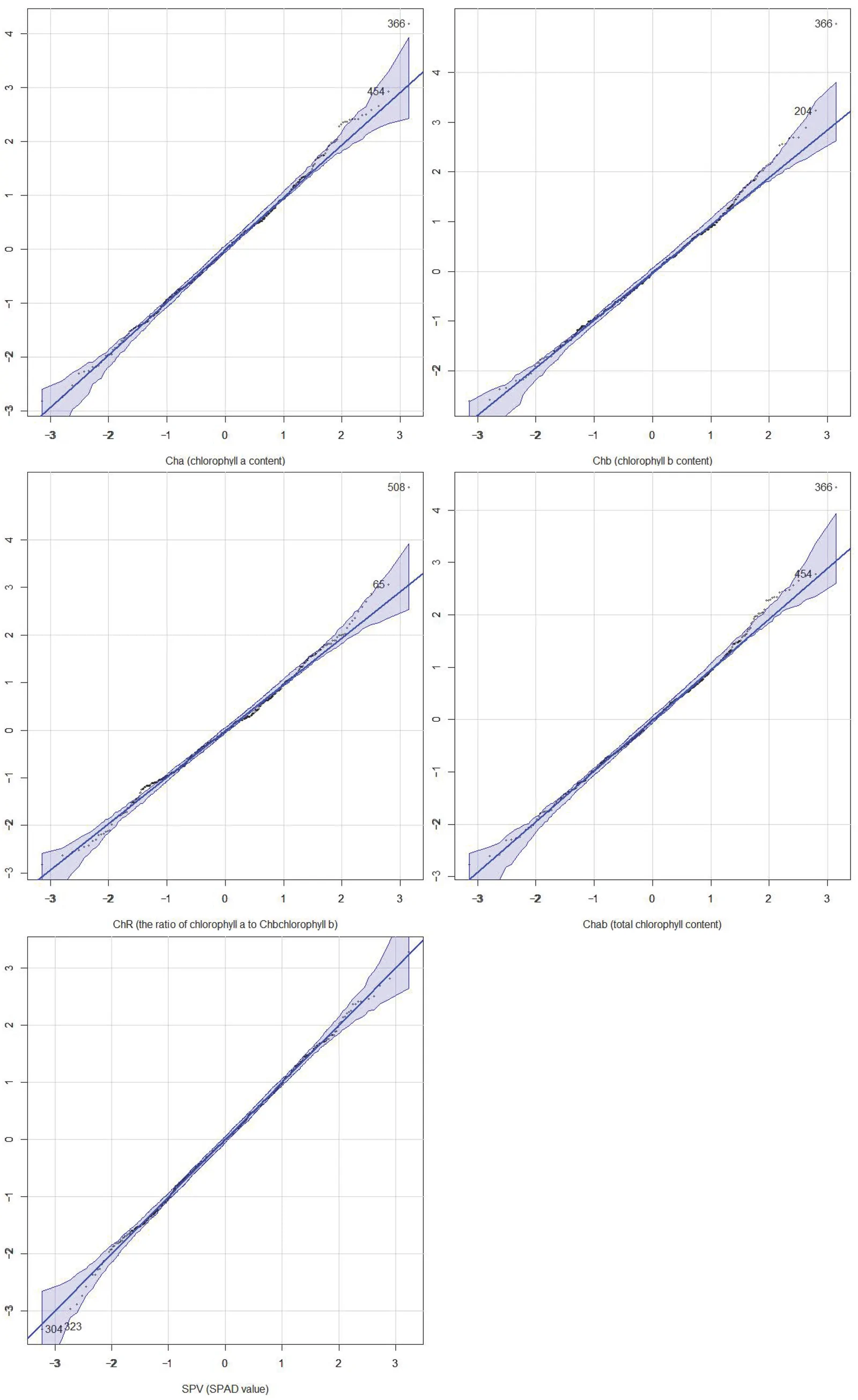
A quantile–quantile (Q-Q) plot of normal distribution for the phenotypic data of the five chlorophyll-related traits across four environments. The four environments were H1 (Wengyuan, June–July 2023), H2 (Wengyuan, September 2023), H3 (Zhanjiang, September 2023), and H4 (Wengyuan, July–August 2022). The panels show (left to right, top to bottom): chlorophyll a (Cha, mg∙dm^−2^), chlorophyll b (Chb, mg∙dm^−2^), chlorophyll ratio Cha/Chb (ChR, dimensionless), total chlorophyll Cha+Chb (Chab, mg∙dm^−2^), and SPAD value (SPV, dimensionless). Cha, Chb, ChR and Chab were not measured in H4 because the analytical acetone required for pigment extraction was temporarily unavailable under tightened precursor-chemical procurement rules; only the non-destructive SPAD value (SPV) was recorded in that environment. The blue lines represented the theoretical quantile line (*y* = *x*), and the blue shaded areas indicated the 95% confidence interval.

Phenotypic evaluations were conducted on the same 219-accession panel across four environments (H1–H4) as described in Section 4.1. For each of the nine traits, the distributions, normality, and variance homogeneity were assessed to determine the appropriate statistical tests for cross-environment comparisons (Table 1; Figure 1 and Figure 2).

**Figure 3:**
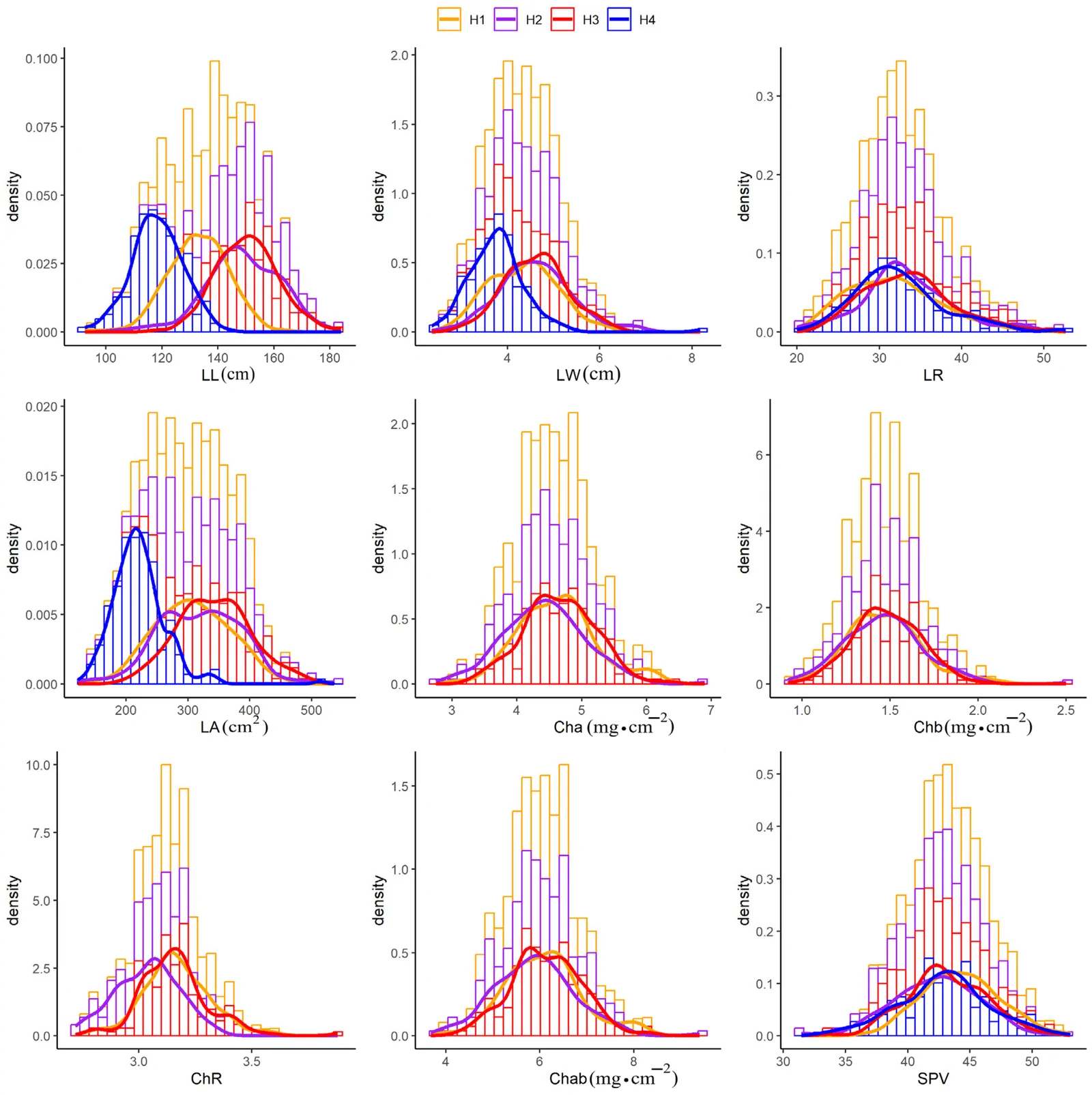
The density distributions for the 219 materials of nine traits across four environments. The four environments were H1 (Wengyuan, June–July 2023), H2 (Wengyuan, September 2023), H3 (Zhanjiang, September 2023), and H4 (Wengyuan, July–August 2022). The panels show (left to right, top to bottom): leaf length (LL, cm), leaf width (LW, cm), leaf ratio LW/LL (LR, dimensionless), leaf area (LA, cm^2^), chlorophyll a (Cha, mg∙dm^−2^), chlorophyll b (Chb, mg∙dm^−2^), chlorophyll ratio Cha/Chb (ChR, dimensionless), total chlorophyll Cha+Chb (Chab, mg∙dm^−2^), and SPAD value (SPV, dimensionless). Cha, Chb, ChR and Chab were not measured in H4 because the analytical acetone required for pigment extraction was temporarily unavailable under tightened precursor-chemical procurement rules; only the non-destructive SPAD value (SPV) was recorded in that environment. Bar-shaped blocks represent density histograms of observed data, and colored lines represent fitted density curves for environments H1 (orange), H2 (purple), H3 (red), and H4 (blue).

Across environments, leaf length (LL), leaf width (LW), leaf length-to-width ratio (LR), and leaf area (LA) deviated from normality (Shapiro–Wilk *p* < 0.05; supported by quantile–quantile (Q-Q) plots; Table 1, Figure 1), with skewness ranging from −0.31 to 1.73 and kurtosis ranging from −0.49 to 9.81 (Table S2). LL approached normality in H4 and LA in H2. Among chlorophyll traits, chlorophyll a content (Cha), total chlorophyll content (Chab), and SPAD value (SPV) were nearer normal in H3/H4 (Shapiro–Wilk *p* > 0.05 across environments), whereas the ratio of chlorophyll a to chlorophyll b (ChR) was mildly skewed (skewness ranging from −0.11 to 0.78). All nine traits showed continuous variation, consistent with polygenic quantitative inheritance and suitability for GWAS (Figure 3).

**Figure 4:**
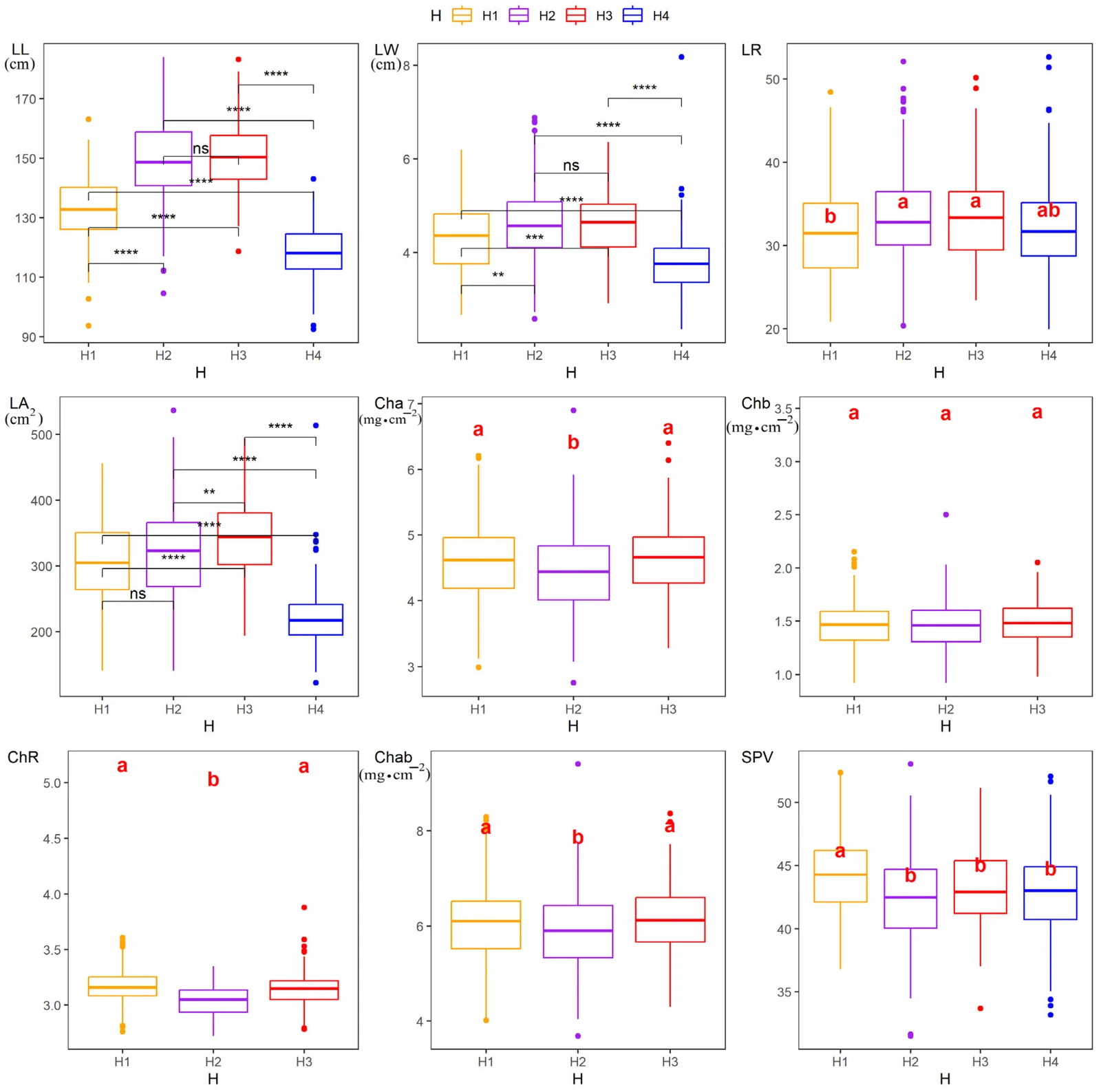
Boxplots of the nine traits across four environments. The four environments were H1 (Wengyuan, June–July 2023), H2 (Wengyuan, September 2023), H3 (Zhanjiang, September 2023), and H4 (Wengyuan, July–August 2022). The panels show (left to right, top to bottom): leaf length (LL, cm), leaf width (LW, cm), leaf ratio LW/LL (LR, dimensionless), leaf area (LA, cm^2^), chlorophyll a (Cha, mg∙dm^−2^), chlorophyll b (Chb, mg∙dm^−2^), chlorophyll ratio Cha/Chb (ChR, dimensionless), total chlorophyll Cha+Chb (Chab, mg∙dm^−2^), and SPAD value (SPV, dimensionless). Cha, Chb, ChR and Chab were not measured in H4 because the analytical acetone required for pigment extraction was temporarily unavailable under tightened precursor-chemical procurement rules; only the non-destructive SPAD value (SPV) was recorded in that environment. ** *p* < 0.01, *** *p* < 0.001, **** *p* < 0.0001; ns, no significance (pairwise comparisons using Wilcoxon tests for LL, LW, and LA). Different small letters denote significant diversity (*p* < 0.05, multiple comparisons with least significant difference (LSD) tests for LR, Cha, Chb, ChR, Chab, and SPV). The boxes represent median and IQR; whiskers indicate the data ranges; dots are outliers from the total materials (*n* = 219).

Because variance homogeneity differed by trait (Levene test, Table 1), Wilcoxon pairwise tests were used for LL, LW, and LA; least significant difference (LSD) tests were used for LR, Cha, Chb (chlorophyll b content), ChR, Chab, and SPV (Levene test, Table 1). LL and LW were significantly larger in H2 and H3 than in H1 or H4 (*p* < 0.05). LA followed the same trend and was significantly higher in H3 than H2, although LL/LW did not differ between those two sites. Cha, Chab and SPV peaked in H1 (early growth stage) and declined later (*p* < 0.05; Figure 4 and Table S2). Chb showed no significant environment pair-effect after correction. These patterns indicate clear stage- and site-dependent shifts in leaf size and chlorophyll status.

### 2.2. Inter-Trait Correlation, Genotypic Effects, and Broad-Sense Heritability (h_B_^2^)

**Figure 5:**
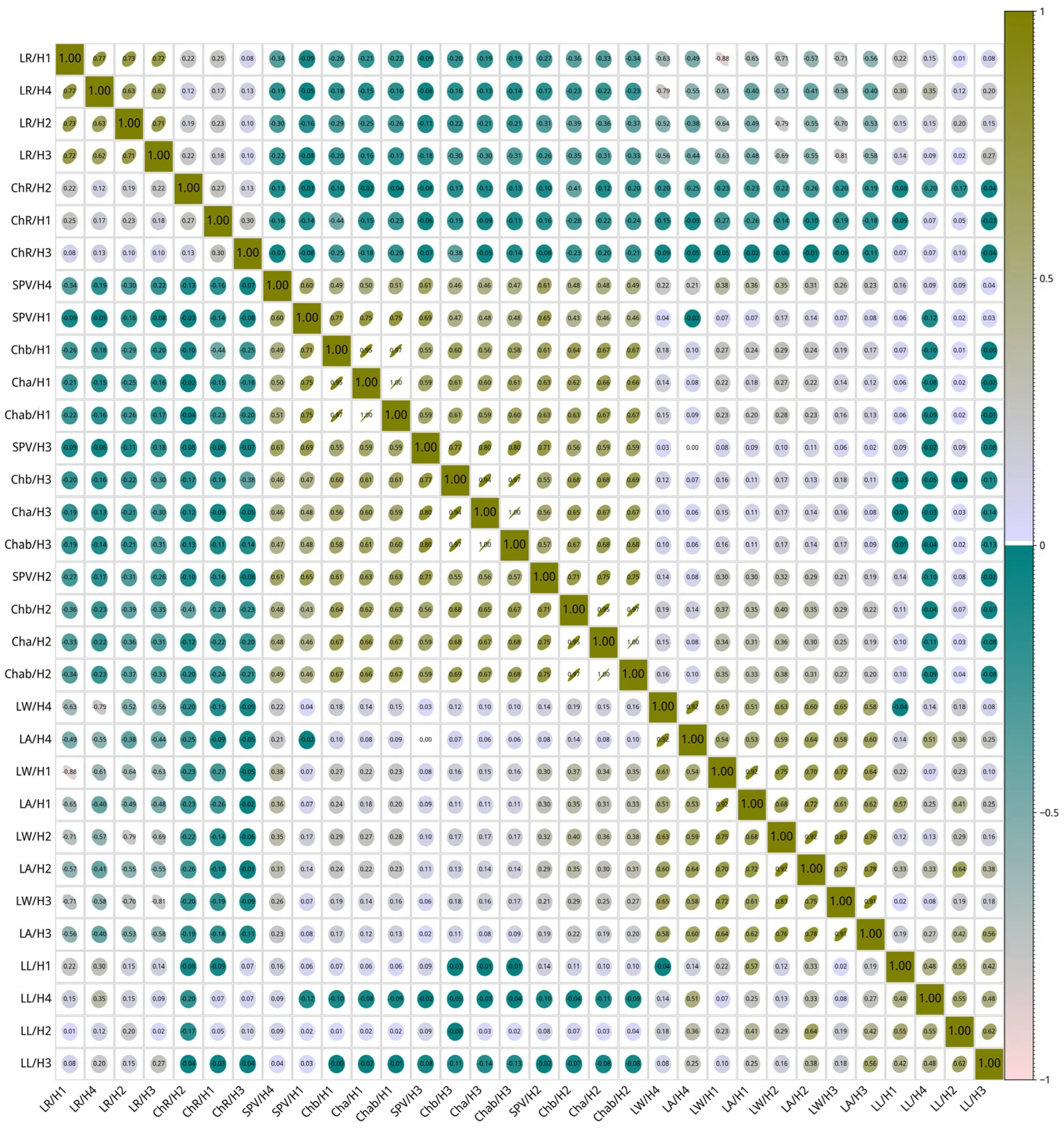
A Pearson correlation matrix for the pairwise relationships among the nine traits across four environments. The four environments were H1 (Wengyuan, June–July 2023), H2 (Wengyuan, September 2023), H3 (Zhanjiang, September 2023), and H4 (Wengyuan, July–August 2022). Cha, Chb, ChR and Chab were not measured in H4 because the analytical acetone required for pigment extraction was temporarily unavailable under tightened precursor-chemical procurement rules; only the non-destructive SPAD value (SPV) was recorded in that environment. Ellipses indicate correlation strength, with colors representing the Pearson correlation coefficient (scale: −1 to 1; yellow-green-cyan gradient). Numerical values denote correlation coefficients; the diagonal represents each trait against itself (r = 1.00).

Pearson correlations separated the nine traits into two blocks (Figure 5): leaf morphology (LL, LW, LR, LA) and chlorophyll (Cha, Chb, ChR, Chab, SPV). Within-block correlations were stronger than cross-block ones (|r| mostly < 0.30 across blocks). Morphology: LR was negatively correlated with LW and LA (−0.41 to −0.89); LA was positively correlated with LL and LW (0.17 to 0.93), while LW explained more LA variance than LL (correlation coefficients between LW and LA ranging from 0.55 to 0.93). Chlorophyll: Cha, Chb, Chab, SPV were mutually positive (0.46 to1.00); ChR was negative to all four (strongest vs. Chb in H1: r = −0.43).

The per-trait correlations between environments were positive and significant (Figure 5), showing temporal stability of ranking.

Analysis of Variance (ANOVA) detected highly significant genotypic differences for all traits in all environments except ChR in H2 (Table 2 and Table 3). Broad-sense heritability (h_B_^2^) averaged 0.47 (LL), 0.61 (LW), 0.58 (LR), 0.67 (LA), 0.53 (Cha), 0.48 (Chb), 0.53 (Chab), and 0.54 (SPV); ChR was low (0.26 overall; 0.18 in H1, 0.35 in H3) (Figures S1 and S2). Except for ChR, traits were mainly genotype-driven, justifying SNP-based dissection.

### 2.3. GWAS Detected 308 Stable SNPs Across 97 Intervals for Nine Traits

Using the same 5,964,084 SNPs and GEMMA linear mixed model (LMM) as in our previous studies [14,20,24], GWAS were performed for each trait in each environment. Significance threshold was set at *p* ≤ 1.5 × 10^−6^ (Bonferroni on 670,149 independent SNPs, r^2^ < 0.2), and a SNP was retained only if significant in two or more environments.

**Figure 6:**
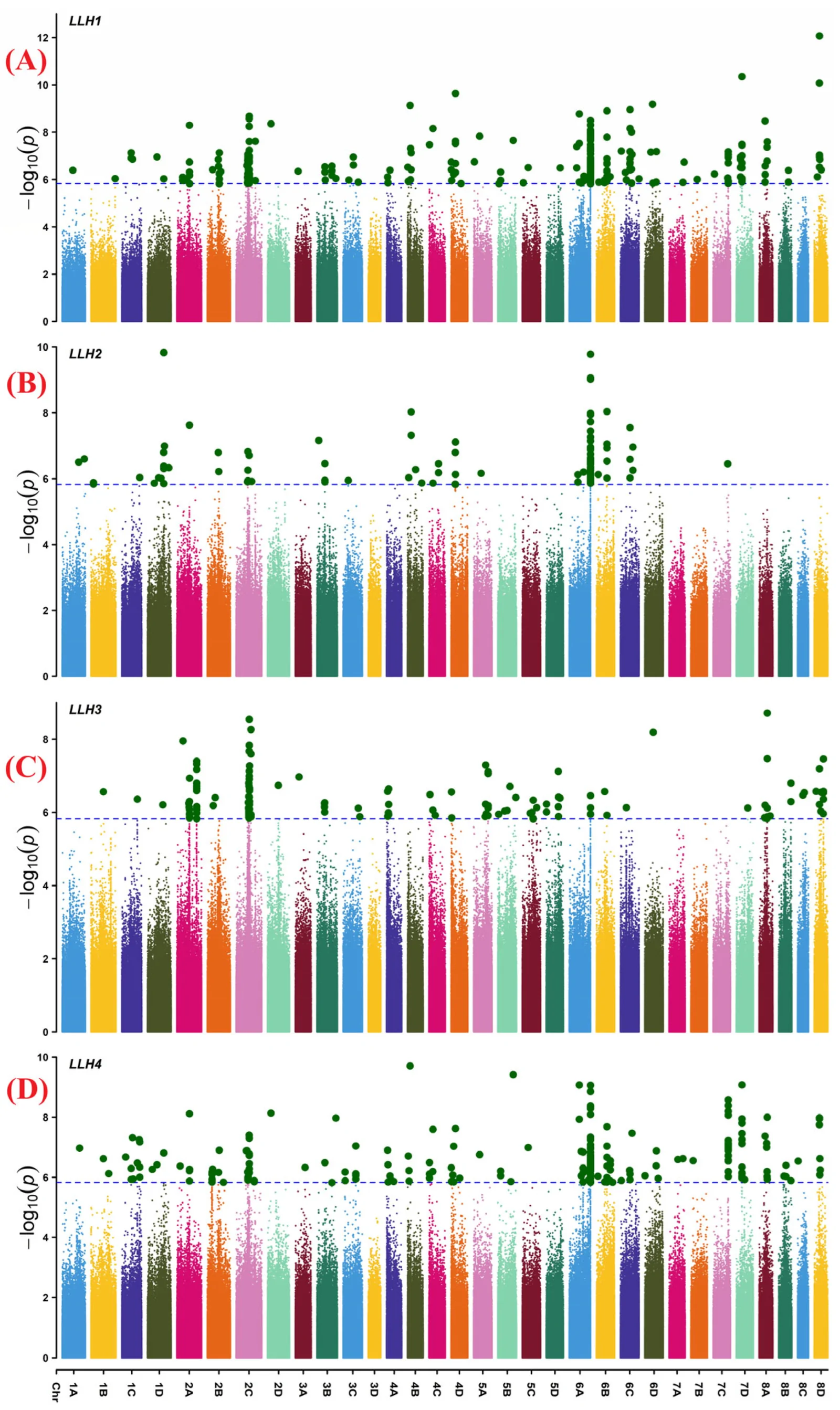
Manhattan plots of the GWAS results for leaf length (LL) across the four environments. (**A**) H1 (Wengyuan, June–July 2023); (**B**) H2 (Wengyuan, September 2023); (**C**) H3 (Zhanjiang, September 2023); (**D**) H4 (Wengyuan, July–August 2022). Each point represents a SNP; the *y*-axis shows −log_10_ (*p*-value) from GEMMA LMM. The blue dashed line indicates the genome-wide significance threshold (*p* = 1.5 × 10^−6^). Chromosome numbers are shown on the *x*-axis. Colored dots denote SNPs on different chromosomes; green dots highlight significant loci above the threshold (blue dashed line, −log_10_(*p*)).

In total, 308 SNPs passed and were collapsed into 97 GWAS-associated intervals (20 kb window) on 30 of 32 chromosomes (absent on 1A, 3D; Table S3, Figure 6 and Figures S6–S14). Of 114 peak SNPs, 64 (56.1%) tagged ≥ 2 traits, suggesting pleiotropy/tight linkage. Per-trait counts and phenotypic variance explained (PVE) were as follows: 165 SNPs and 10.59–20.89% for LL, 82 and 10.34–19.77% for LW, 35 and 10.55–15.24% for LR, 53 and 10.34–16.40% for LA, 35 and 11.26–16.36% for Cha, 34 and 10.21–15.37% for Chb, 73 and 10.12–19.62% for ChR, 35 and 11.05–16.15% for Chab, and 168 and 10.43–17.77% for SPV.

### 2.4. Candidate Gene Prioritization from 97 GWAS-Associated Intervals

Merging 308 SNPs into 97 GWAS-associated intervals yielded 98 predicted genes (52 intervals contained genes, and nine genes were functionally uncharacterized). In total, 60 intervals were multi-trait (up to four intervals encompassing all nine traits). Using AP85-441 annotation and literature, 20 genes in 16 intervals were prioritized as photosynthesis-relevant and categorized into four groups according to their regulatory pathways (Table 4; Table S4). The remaining 78 predicted genes and nine unknown-function hits are listed in Table S4 for follow-up.

## 3. Discussion

### 3.1. Complementarity with Previous GWAS and Characterization of the Sugarcane Panel

The present study and our recent GWAS on leaf photosynthetic gas exchange parameters [24] represent complementary components of a long-term initiative to enhance photosynthetic performance in sugarcane. While our previous work focused on physiological outputs—specifically intercellular CO_2_ concentration, net photosynthetic rate, transpiration rate, and stomatal conductance—the current study dissects the structural and pigment-based determinants of photosynthesis, including four leaf morphology traits (LL, LW, LR, and LA) and five chlorophyll-related traits (Cha, Chb, ChR, Chab, and SPV). These traits constitute the photosynthetic structural foundation and determine the light-harvesting capacity of leaves, directly governing light capture, chloroplast development, and energy conversion. However, it is important to note that they are indirect proxies of photosynthesis and do not directly measure photosynthetic efficiency.

Genetically, the two studies revealed largely independent architectures. Only two genes, *Sspon.02G0019170-1A* (encoding a WRKY transcription factor) and *Sspon.02G0019190-3C* (encoding the UV-B receptor UVR8), were co-detected. Functionally, both are linked to stress adaptation rather than the core machinery of carbon assimilation. This genetic independence is corroborated by phenotypic correlation analyses, which showed uniformly low coefficients (|r| < 0.30; Table S5) between gas exchange parameters and leaf morphology/chlorophyll traits across all environments. Consequently, presenting these datasets separately prevents the obscuring of distinct biological interpretations and respects the complexity of the sugarcane polyploid genome.

From a breeding perspective, the two studies also serve different practical purposes. The leaf morphology and chlorophyll-related traits identified here are highly amenable to high-throughput phenotypic screening, making them ideal targets for large-scale variety evaluation and early-generation selection. In contrast, gas exchange parameters require precise, instrument-based measurements under controlled conditions and are better suited to precision physiological identification and in-depth functional validation [24]. Together, these independent investigations provide a comprehensive genetic framework for improving the photosynthetic traits of sugarcane, but the current results do not directly demonstrate enhanced photosynthetic efficiency and should be validated in combination with gas exchange or yield trials in future studies.

The diversity of the panel further supports the robustness of our findings. The 219 elite materials analyzed here, originally described in our earlier studies [14,20], represent germplasm from more than 13 countries or regions (Table S6). A significant proportion (74.4%) were released post-1960, and 163 materials trace back to 21 globally recognized backbone parents, including CP49-50, Co419, and NCo310—foundational genotypes in Chinese sugarcane breeding. Notably, several candidate genes identified herein, such as *Sspon.01G0024470-3C*, *Sspon.01G0024480-3C*, *Sspon.06G0023090-1B*, and *Sspon.07G0019440-2C*, have also been implicated in cane yield and fiber content within the same population [14,20], underscoring their potential pleiotropic effects on agronomically important traits.

### 3.2. Phenotypic Relationships and Heritability of Photosynthesis-Related Traits

Comparisons across environments showed that LL, LW, and LA were all significantly larger in H2 (Wengyuan September) and H3 (Zhanjiang September) than in H1 or H4. This could be because in both Wengyuan and Zhanjiang, sugarcane plants were taller and had longer and wider leaves in September compared to June and July. Furthermore, LA was significantly higher in H3 than H2, although LL/LW did not differ between those two sites. The reason could be that plant growth in Zhanjiang was more rapid than in Wengyuan during the same period (September), due to greater sunlight exposure and higher temperatures.

Pearson correlation analysis resolved the nine traits into two distinct clusters: leaf morphology and chlorophyll-related traits. Cross-cluster correlations were consistently weaker than within-cluster values, indicating limited phenotypic coupling despite their shared contribution to photosynthesis.

Within the leaf morphology cluster, leaf width (LW) exerted a stronger influence on leaf ratio (LR) and leaf area (LA) than leaf length (LL). Specifically, LW was negatively correlated with LR but positively correlated with LA, whereas LR and LA exhibited a strong negative correlation. This suggests that sugarcane leaf area is more sensitive to variations in width than in length. Similar patterns have been observed in rice, where flag leaf area correlates more strongly with length than width [7].

Among chlorophyll-related traits, Cha, Chb, Chab, and SPV were strongly positively correlated, reflecting their shared biosynthetic pathways. In contrast, the chlorophyll a/b ratio (ChR) showed negative correlations with these traits, indicating that the regulation of chlorophyll stoichiometry is partially decoupled from total chlorophyll accumulation. This aligns with observations in soybean, where the heritability and correlation patterns of the chlorophyll a/b ratio differ markedly from those of total chlorophyll content [25].

Broad-sense heritability (h_B_^2^) estimates were high for most traits, with the notable exception of ChR. This reduced heritability likely arises from the mathematical nature of the ratio: if Cha and Chb change inversely, ChR varies substantially even with minor changes in absolute values, inflating the mean square error for blocks relative to that for genotypes. Conversely, the high h_B_^2^ observed for LR was attributable to its high coefficient of variation across environments. Interestingly, despite its low heritability, ChR yielded the largest number of significant SNPs (73). This is likely due to the high stability of ChR across environments (mean values ranging from 3.04 to 3.17), which facilitated the detection of consistent SNP signals across trials (Figure S12). While this pattern might initially suggest inflated false positives, the uniformly low genomic inflation factors (λ = 1.027–1.034, Table S7) across ChR environments argue against population stratification as the primary driver. A more plausible interpretation is that ChR is governed by a complex genetic architecture with substantial non-additive effects or strong G×E interactions, which would limit the heritability captured by the additive LMM while still producing detectable association signals at individual loci. Nevertheless, this inference remains tentative and requires validation in independent panels or through experimental verification of candidate genes. Although separate ANOVAs were conducted for each environment, the high and significant positive correlations observed among environments for each trait (Figure 5) suggested that genotype effects predominate over G×E interactions. In addition, due to the focus of this study on GWAS rather than stability analysis, a sub-environment GWAS strategy was adopted, which is consistent with our previous GWAS.

Additionally, all phenotypic measurements were taken on the +2 leaf of plants at the elongating stage (approximately four to six months after ratooning), corresponding to the active vegetative growth phase when leaf morphology and chlorophyll content are most relevant to photosynthetic capacity. While H1 and H4 (June–August) represent the early elongating stage and H2/H3 (September) represent the peak elongating stage, the consistent genetic correlations across environments (Figure 5) indicate that genotype effects predominate over stage-specific variation. Nevertheless, we acknowledge that chronological age and developmental stage were partially confounded with environment, which may contribute to phenotypic differences among H1–H4.

### 3.3. Candidate Genes and Functional Implications

Based on functional annotation and literature mining, 20 key candidate genes were potentially associated with leaf photosynthesis.

The candidate gene *Sspon.04G0001350-4D* encodes monogalactosyldiacylglycerol (MGDG) synthase, while *Sspon.04G0032010-2D* and *Sspon.04G0032040-2D* both encode the Orange (OR) protein, and *Sspon.07G0020110-3C* encodes the chloroplast stem-loop binding protein of 41 kDa b (CSP41b). Previous studies have consistently demonstrated that MGDG [26], OR [27], and CSP41b [28] are closely involved in chloroplast biogenesis and development, processes that are fundamental to efficient photosynthesis. The candidate gene *Sspon.07G0020090-1P* encodes a PsbP domain-containing (PPD) protein. Members of the PsbP protein family are well recognized as essential components of the photosynthetic apparatus and play critical roles in maintaining photosystem II activity and overall photosynthetic performance in plants [29].

Several additional candidate genes identified in this study encode proteins previously implicated in leaf growth and development. *Sspon.01G0015510-3D* and *Sspon.04G0005720-2B* encode ADP-ribosylation factors, *Sspon.04G0001230-1A* encodes an Aux/IAA protein, *Sspon.06G0023090-1B* encodes an F-box protein, and *Sspon.07G0019440-2C* encodes a MYB transcription factor. Consistent with our findings, earlier studies have demonstrated that ADP-ribosylation factors [7], Aux/IAA proteins [30], F-box proteins [31], and MYB transcription factors [31,32] play pivotal roles in leaf morphogenesis, expansion, and developmental regulation.

Other candidate genes identified here are linked to a broader range of chloroplast function, photosynthesis, and environmental responses. *Sspon.01G0024470-3C* encodes a RmlC-like cupin superfamily protein; *Sspon.01G0024480-3C* encodes Memb11; *Sspon.01G0015500-3D* encodes an ATP-dependent Clp protease proteolytic subunit; *Sspon.02G0019170-1A* encodes a WRKY transcription factor; *Sspon.02G0019190-1A* and *Sspon.02G0019190-3C* encode UVR8; *Sspon.03G0011650-2B*, *Sspon.03G0015430-2B*, and *Sspon.08G0012770-3D* encode pentatricopeptide repeat-containing (PPR) proteins; and *Sspon.04G0001770-2B* encodes a protein kinase. Previous research has linked RmlC-like cupin domain superfamily [33], Memb11 [34], WRKY transcription factors [35], UVR8 [36], ATP-dependent Clp proteases [5], PPR proteins [37], and protein kinases [7] not only to leaf development but also to chloroplast development, photosynthetic regulation, and ultraviolet protection.

Notably, strong supporting evidence emerged from cross-referencing our previous GWAS on yield, fiber content, and gas exchange parameters. For instance, *Sspon.01G0024470-3C* and *Sspon.01G0024480-3C* have been linked to cane yield [14] and fiber content [20], respectively. Furthermore, *WRKY55* and *UVR8* were also detected in our previous analysis of gas exchange parameters, albeit primarily associated with stress responses [24]. Several other candidates align with known functions in model crops. Protein kinases and MYB transcription factors identified here have previously been associated with cane yield, plant height, and stalk diameter [14], as well as leaf angle [23]. Similarly, ADP-ribosylation factors have been implicated in leaf length regulation in rice [7], while ATP-dependent Clp proteases control leaf width in alfalfa [5]. These consistencies reinforce the biological relevance of our candidate list. Collectively, ten genes stand out as particularly promising candidates prioritized for future functional validation (Table S8). These include *Sspon.01G0024480-3C* (Chromosome 1C, encoding Memb11, associated with LW and SPV) and *Sspon.01G0024470-3C* (Chromosome 1C, encoding a RmlC-like cupin protein, associated with LW and SPV). Other high-priority candidates include *Sspon.04G0001230-1A* (*IAA9*, affecting multiple traits), *Sspon.04G0001770-2B* (protein kinase, associated with chlorophyll traits), and *Sspon.08G0012770-3D* (PPR protein, associated with LR). These genes represent a valuable hypothesis-generating resource for future functional validation, bridging the gap between genetic dissection and molecular breeding for enhanced photosynthetic performance.

In addition, sugarcane cultivars are highly complex polyploids (2n ≈ 100–130). In this study, SNPs were filtered to biallelic sites against the haploid *S. spontaneum* reference, a common workaround that permits standard diploid-oriented GWAS tools but effectively diploidizes the genome [14,23]. This approach ignores allele dosage, which is a major driver of phenotypic variation in polyploids. By collapsing multi-allelic, multi-copy variation into a binary code, we likely miss loci whose effects are dominated by dosage and underestimate additive genetic variance and PVE. Consequently, the 308 significant SNPs and 98 candidate genes reported here represent a conservative subset of the true polyploid association landscape. This limitation is shared by essentially all current sugarcane GWAS based on linear-reference biallelic SNPs and does not invalidate the biological plausibility of the candidates, but it bounds the resolution of the genetic architecture we can claim.

It should be noted that candidate gene prediction within 20 kb GWAS-associated intervals is inherently a hypothesis-generating step. Without transcriptomic or proteomic data in the same panel, the causal genes cannot be definitively identified. However, the 20 kb window is consistent with the estimated linkage disequilibrium (LD) decay distance in sugarcane [14,20,24], and functional annotation of predicted genes was cross-referenced with the published literature in model crops. This approach represents a standard first step in crop GWAS, and the prioritized candidates warrant future validation via expression analysis or targeted mutagenesis.

## 4. Materials and Methods

### 4.1. Materials and Experimental Design

Field trials were originally established in 2019 (Wengyuan) and 2020 (Zhanjiang) using the same 219 materials, and genomic DNA for resequencing was extracted from these plantings [14,20,24]. Planting was carried out on 26 February 2019 at Wengyuan and on 18 February 2020 at Zhanjiang. At both sites, the experiments followed a randomized complete block design with three replicates (blocks). Each plot consisted of three rows spaced 110 cm apart, with 16 plants per row and an intra-row spacing of 25 cm. After disinfection, stalks were cut into single-bud setts, raised in seedling nurseries, and transplanted to the field when buds reached approximately 20 cm in height. At both sites, sugarcane was harvested in December each year, followed immediately by ratooning trials in January of the next year. In each year, field management at both locations followed standard local agronomic practices, including routine fertilization; irrigation; and disease, pest, and weed control.

For the present study, the phenotypic data of the nine traits were collected from the ratoon crops of these same trials during the 2022 and 2023 growing seasons, corresponding to four environments: H4 (Wengyuan, July–August 2022) corresponded to the third ratoon (R3) of the 2019 planting; H1 and H2 (Wengyuan, June–July and September 2023, respectively) corresponded to the fourth ratoon (R4) of the same 2019 planting; H3 (Zhanjiang, September 2023) corresponded to the third ratoon (R3) of the 2020 planting. All measurements were taken on the +2 fully expanded leaf during the elongating stage. We acknowledge that H1/H2 (R4, Wengyuan) and H3 (R3, Zhanjiang) differ not only in site and measurement month but also in ratoon order, so crop-cycle age is partially confounded with site and season. However, the high genotype-level correlations across environments (Figure 5) indicate that the genetic rank of the 219 materials is largely preserved across ratoon cycles, and the GWAS was performed separately per environment to avoid forcing a single additive G+E model on heterogeneous ratoon stages.

### 4.2. Statistical Analysis of Phenotypic Data

The nine traits evaluated in this study were selected based on their established contributions to sugarcane photosynthetic capacity and biomass accumulation. Leaf morphology traits (LL, LW, LR, LA) determine the light-harvesting surface area and canopy architecture, directly influencing radiation interception. Chlorophyll traits (Cha, Chb, ChR, Chab, SPV) represent the pigment apparatus responsible for light absorption and energy transfer. Together, these traits constitute the structural and biochemical foundation of leaf photosynthesis, and have been widely used as proxies for photosynthetic capacity in crops [4,6].

Four leaf morphology traits were evaluated: LL, LW, LR, and LA. LL was measured from the base to the tip of the +2 leaf, after which LW was recorded at the widest point of the same leaf. LR was calculated as LL/LW. LA was determined using a handheld portable leaf area meter (YMJ-B; Hangzhou Top Instrument Co., Ltd., Hangzhou, China).

Five chlorophyll-related traits were also assessed, including Cha, Chb, ChR, Chab, and SPV. SPV was measured with a portable SPAD meter (SPAD-502 Plus; Konica Minolta, Tokyo, Japan). The detailed methods for determining Cha, Chb, and Chab followed the procedures described previously [38], and ChR was calculated as Cha/Chb. Notably, in environment H4, acetone for chlorophyll extraction [38] was not available because the institutional procurement of Class III precursor chemicals (acetone) was suspended under updated public-security filing rules; consequently Cha, Chb, ChR and Chab were not obtained for H4, whereas SPV was still measured non-destructively.

The normality of the phenotypic distributions across environments was assessed using the Shapiro–Wilk test (Table 1) and Q-Q plots (Figure 1 and Figure 2). Each dataset (219 clones × three or four environments) comprised the mean value of three replicates (blocks) for each clone in each environment. The tests for all traits were performed independently. Homogeneity of variance was evaluated using Levene’s test. For each trait, if the Levene test showed a *p*-value below 0.05 (Table 1), the trait was considered to violate the assumptions of parametric ANOVA, and pairwise comparisons among environments were performed using Wilcoxon tests. Otherwise, multiple comparisons were conducted using LSD tests. All tests, comparisons, and Pearson correlation analysis were performed in R (version 4.3.2; R Foundation, Vienna, Austria; www.r-project.org, accessed on 31 December 2023), which was also used for all graphical visualizations. Additionally, a separate ANOVA was conducted for each trait within each individual environment, with the model including the effects of material (genotype) and block (replicate). The broad-sense heritability (h_B_^2^) of each trait was calculated by the method of ANOVA: h_B_^2^ = δ_g_^2^/(δ_g_^2^ + δ_e_^2^/r) [39].

### 4.3. GWAS

Paired-end sequencing was conducted on an Illumina Hiseq2500 platform by Beijing Novogene Bioinformatics Technology Co., Ltd. (Beijing, China), achieving an average sequencing depth of approximately 21× for *S. spontaneum*. The analysis began by removing adapter sequences and low-quality reads from the raw paired-end data of each sample. Subsequently, the cleaned reads were aligned to the reference genome AP85-441 using the bwa software (version 0.7.17; Broad Institute, Cambridge, MA, USA) with default parameters. Uniquely mapped reads with correct insert sizes were retained for subsequent analysis. SNPs were called by GATK (version 4.1; Broad Institute, Cambridge, MA, USA). The SNPs were filtered using the following parameters: call quality divided by depth (QD) < 2.0; mapping quality (MQ) < 40.0; Fisher’s exact test (FS) > 60.0; SOR (Strand Odds Ratio) > 3.0; MQRankSum < −12.5; and ReadPosRankSum < −8.0. Then, using PLINK software (version 1.07; Shaun Purcell, Center for Human Genetic Research, Massachusetts General Hospital, Boston, MA, USA), SNPs with a minor allele frequency (MAF) > 0.05 and a missing rate < 0.2 were retained, and SNPs with more than two alleles were removed. A final set of 5,964,084 high-quality SNPs was obtained for downstream analysis.

Population structure was evaluated using principal component analysis (PCA) and a kinship matrix calculated from the 5,964,084 SNPs. The first 34 principal components explained less than 50% of the total genetic variance [24], and no obvious clustering was observed (Figures S3–S5). The kinship heatmap showed no discrete blocks, indicating low population stratification. GWAS was conducted using GEMMA (version 0.98.5; Zhou and Stephens; Department of Biostatistics, University of Michigan, Ann Arbor, MI, USA) LMM [40]. The K-only LMM approach is consistent with our prior GWAS on the same 219-accession panel and 5,964,084 SNPs [14], where population structure was assessed via ADMIXTURE/PCA but the GWAS itself was conducted with a kinship matrix only. To further validate model adequacy in the present study, genomic inflation factors (λ) were additionally calculated for all trait–environment combinations, which ranged from 1.00251 to 1.039248 (median = 1.028302), with all values close to 1.0 (Table S7, Figures S6–S14), confirming that false positive rates were well controlled without including a covariate matrix. Each association test for each trait in each environment was performed with phenotypic data, which comprised the mean value of three replicates for the 219 materials. The total number of the association tests was 32 (nine traits across four (for LL, LW, LR, LA, and SPV) or three (for Cha, Chb, ChR, and Chab) environments). The commands used to calculate the relatedness matrix and to perform association analysis with PLINK (version 1.07; Shaun Purcell, Center for Human Genetic Research, Massachusetts General Hospital, Boston, MA, USA) binary PED files were “./gemma -bfile [prefix] -gk2 -o [prefix]” and “./gemma -bfile [prefix] -k [filename] -lmm 1 -o [prefix]”, respectively.

To control for false positives, the independent SNPs were identified via PLINK (version 1.07; Shaun Purcell, Center for Human Genetic Research, Massachusetts General Hospital, Boston, MA, USA) with the parameter “--indep-pairwise 100 50 0.2.” A total of 670,149 independent SNPs were retained with r^2^ ≥ 0.2, and the significance threshold was set at *p* ≤ 1/670,149 = 1.5 × 10^−6^, which was used to avoid false negatives [14,20,24]. Manhattan and Q-Q plots were generated using the “CMplot” package in R (version 4.3.2; R Foundation, Vienna, Austria; www.r-project.org, accessed on 31 December 2023).

### 4.4. Identification of Candidate Genes

In this study, GWAS-significant SNPs were defined as those with *p* ≤ 1.5 × 10^−6^ in the LMM in two or more environments. These are listed as the “Number of GWAS-significant SNPs” for each trait–locus combination in Table S3. GWAS-associated loci (intervals) were defined as genomic regions associated with a trait, each designated by a locus name (e.g., *qLL1C_1* on chromosome 1C in Table S3). Based on our previous studies [14,20,24], each locus represents a contiguous interval defined by the boundaries of the LD block (r^2^ ≥ 0.2) or ± 20 kb around the most significant SNP, whichever was larger. Within each locus (interval), the SNP with the smallest *p* value was designated as the peak SNP (representative SNP). The peak SNP is reported with its physical position, alleles, minor allele frequency (MAF), *p* value, and phenotypic variance explained (PVE; %). For example, in Table S3, the peak SNP for *qLL1C_1* is located at position 46,526,064 (A/G, MAF = 0.06, *p* = 7.37 × 10^−8^, PVE = 12.42%). Associated SNPs located within genes or intergenic regions were identified by aligning sequencing data to the *Saccharum spontaneum* reference genome (AP85-441) [41]. Marker positions and corresponding gene annotation information were extracted accordingly (Table S4). The term QTL is used only when referring to previously reported quantitative trait loci from linkage or QTL mapping studies.

## 5. Conclusions

This study provides the first comprehensive GWAS dissection of leaf morphological and chlorophyll-related traits in sugarcane. We delineated the genetic architecture of nine photosynthesis-related traits and identified 308 GWAS-significant SNPs within 97 GWAS-associated intervals, which harbor 98 predicted genes. Functional annotation highlighted 20 genes within 16 intervals, potentially associated with leaf photosynthesis. These candidates are primarily involved in pathways related to ADP-ribosylation factors, ATP-dependent Clp protease proteolytic subunits, protein kinases, F-box proteins, MYB and WRKY transcription factors, UVR8, pentatricopeptide repeat-containing (PPR) proteins, Aux/IAA proteins, MGDG synthase, OR protein, PsbP domain-containing (PPD) proteins, and CSP41b. Based on prior evidence, ten genes—*Sspon.01G0024480-3C*, *Sspon.01G0024470-3C*, *Sspon.01G0015510-3D*, *Sspon.04G0005720-2B*, *Sspon.01G0015500-3D*, *Sspon.04G0001230-1A*, *Sspon.04G0001770-2B*, *Sspon.06G0023090-1B*, *Sspon.07G0019440-2C*, and *Sspon.08G0012770-3D*—emerged as particularly promising candidates underlying these traits.

It is important to note that the traits analyzed here (leaf morphology and chlorophyll content) are indirect proxies of photosynthesis, distinct from direct gas exchange parameters such as net photosynthetic rate, stomatal conductance, and transpiration. In our previous GWAS on the same panel, we reported loci for gas exchange traits; however, phenotypic correlations between the two sets of traits are generally weak (|r| < 0.30), indicating largely independent genetic architectures. Therefore, the current results do not directly demonstrate enhanced photosynthetic efficiency but instead offer genetic resources for the photosynthetic structural foundation (light capture and pigment composition). The prioritized candidate genes provide a basis for future studies to combine structural and physiological traits or to validate their effects on actual photosynthetic rates. Collectively, these markers and candidate genes serve as valuable resources for marker-assisted breeding targeting improved photosynthetic traits in sugarcane.

Additionally, this study has several limitations that should be addressed in future work. First, G×E interactions were not explicitly modeled, although the high cross-environment correlations suggest that genetic effects predominate. A comprehensive multi-environment trial with repeated measures across years or sites would enable formal G×E analysis and identification of stable GWAS-associated loci. Second, because H1/H2 and H4 originated from R4 and R3 of the Wengyuan planting, whereas H3 was R3 of the Zhanjiang planting, ratoon order was not fully balanced across environments. Sugarcane canopy architecture, leaf area, and chlorophyll content are known to shift between plant cane and successive ratoons due to changes in shoot/stool density and root architecture. Although we measured at the same elongating stage and observed strong cross-environment genetic correlations, we cannot exclude that part of the H1–H4 mean differences reflects ratoon-stage effects rather than pure site/season effects. A future design with plant cane and successive ratoons of the same materials evaluated in the same year would disentangle crop-cycle from site effects. Third, the candidate genes identified here are predictions based on the GWAS-associated intervals and functional annotation; experimental validation via transcriptomics, gene editing, or transgenic approaches is required to confirm their causal roles. Fourth, the diploid-based SNP calling approach does not capture allele dosage effects inherent to sugarcane’s polyploid genome. Future GWAS incorporating polyploid-aware models and haplotype-resolved assemblies will provide a more complete picture of the genetic architecture of leaf photosynthetic traits in sugarcane.

## Figures and Tables

**Table 1:** *p*-values of Shapiro–Wilk test and Levene test for nine traits across four environments.

Trait	LL (cm)	LW (cm)	LR	LA (cm^2^)	Cha (mg∙dm^−2^)	Chb (mg∙dm^−2^)	ChR	Chab (mg∙dm^−2^)	SPV
Shapiro–Wilk test	1.10 × 10^−3^	5.86 × 10^−5^	1.14 × 10^−7^	3.78 × 10^−5^	0.16	6.00 × 10^−4^	2.00 × 10^−4^	0.05	0.65
Levene test	2.00 × 10^−4^	2.10 × 10^−2^	0.86	8.78 × 10^−10^	0.46	0.27	0.95	0.41	0.18

Cha, Chb, ChR and Chab were not measured in H4 because the analytical acetone required for pigment extraction was temporarily unavailable under tightened precursor-chemical procurement rules; only the non-destructive SPAD value (SPV) was recorded in that environment.

**Table 2:** The results of the ANOVA for the four leaf morphology traits among the materials in four environments.

Environment	Source	Df	MS for the Four Leaf Morphology Traits			
			LL (cm)	LW (cm)	LR	LA (cm^2^)
H1	Block	2	565.60 **	0.09	55.67 **	539.42
	Genotype	197	344.78 **	1.61 **	89.95 **	11,129.13 **
	Error	394	87.17	0.19	9.49	1784.99
H2	Block	2	2596.70 **	430.23 **	5830.67 **	30,105.53 **
	Genotype	183	527.32 **	1.89 **	95.23 **	13,205.00 **
	Error	366	53.56	0.22	20.01	739.28
H3	Block	2	11,017.51 **	10.88 **	53.09 **	210,829.72 **
	Genotype	180	360.22 **	1.39 **	81.98 **	10,574.07 **
	Error	360	90.8	0.17	11.67	1733.97
H4	Block	2	3978.27 **	1.06 **	28.62 **	1971.33 **
	Genotype	187	249.62 **	1.24 **	90.50 **	5890.93 **
	Error	374	170.08	0.51	33.23	1201.47

Df, degrees of freedom; MS, mean square; **, mark significance level at 0.01.

**Table 3:** The results of the ANOVA for the five chlorophyll-related traits among the materials in four environments.

Environment	Source	Df	MS for the Five Traits Related to Leaf Chlorophyll Content				
			Cha (mg∙dm^−2^)	Chb (mg∙dm^−2^)	ChR	Chab (mg∙dm^−2^)	SPV
H1	Block	2	5.65 **	0.31 **	0.63 **	8.19 **	156.86 **
	Genotype	197	1.12 **	0.14 **	0.07 **	2.02 **	27.61 **
	Error	394	0.29	0.04	0.04	0.52	6.11
H2	Block	2	4.18 **	0.73 **	7.11 **	3.97 **	202.44 **
	Genotype	183	1.21 **	0.16 **	0.06	2.21 **	35.85 **
	Error	366	0.22	0.04	0.06	0.4	3.92
H3	Block	2	7.71 **	0.28 **	1.15 **	10.43 **	284.19 **
	Genotype	180	0.93 **	0.11 **	0.07 **	1.63 **	28.27 **
	Error	360	0.23	0.03	0.03	0.4	5.84
H4	Block	2	NA	NA	NA	NA	837.54 **
	Genotype	187	NA	NA	NA	NA	39.05 **
	Error	374	NA	NA	NA	NA	15.14
	Error	374	NA	NA	NA	NA	15.14

Df, degrees of freedom; MS, mean square; **, mark significance level at 0.01; NA, not measured in H4 (acetone unavailable, 2022 field season).

**Table 4:** Predicted key candidate genes related to photosynthesis-related traits and functional annotation.

Group	Symbol	Functional Annotation	Candidate Gene ID
I: Chloroplast Biogenesis	MGD3	“PREDICTED: probable monogalactosyldiacylglycerol synthase 3, chloroplastic [*Setaria italica*]”	*Sspon.04G0001350-4D*
	OR	“protein ORANGE, chloroplastic-like [*Aegilops tauschii* subsp. *tauschii*] “	*Sspon.04G0032010-2D*, *Sspon.04G0032040-2D*
	CSP41B	“PREDICTED: chloroplast stem-loop binding protein of 41 kDa b, chloroplastic-like isoform X1 [*Zea mays*]”	*Sspon.07G0020110-3C*
	PPD4	“PREDICTED: psbP domain-containing protein 4, chloroplastic [*Oryza sativa* Japonica Group]”	*Sspon.07G0020090-1P*
II: Leaf Development	ARF	PREDICTED: ADP-ribosylation factor [*Setaria italica*]	*Sspon.01G0015510-3D*, *Sspon.04G0005720-2B*
	IAA9	IAA9—auxin-responsive Aux/IAA family member [*Zea mays*]	*Sspon.04G0001230-1A*
	-	PREDICTED: F-box protein At3g59000 [*Oryza sativa Japonica Group*]	*Sspon.06G0023090-1B*
	MYB4	transcription factor MYB8 [*Zea mays*]	*Sspon.07G0019440-2C*
III: Stress/Signaling/Chloroplast Quality Control	PCO2	“cupin, RmlC-type [*Zea mays*]”	*Sspon.01G0024470-3C*
	MEMB11	PREDICTED: membrin-11-like [*Oryza brachyantha*]	*Sspon.01G0024480-3C*
	CLPP4	ATP-dependent Clp protease proteolytic subunit [*Zea mays*]	*Sspon.01G0015500-3D*
	WRKY55	PREDICTED: probable WRKY transcription factor 41	*Sspon.02G0019170-1A*
	UVR8	Ultraviolet-B receptor UVR8 [*Dichanthelium oligosanthes*]	*Sspon.02G0019190-1A*, *Sspon.02G0019190-3C*
	CRR21 (homolog of *At4g01990* and *At3g25210*)	“PREDICTED: pentatricopeptide repeat-containing protein *At5g55740*, chloroplastic [*Zea mays*]/*At4g01990*, *At3g25210*, mitochondrial-like [*Setaria italica*]”	*Sspon.03G0011650-2B*, *Sspon.03G0015430-2B*, *Sspon.08G0012770-3D*
IV: Other Regulators	-	Protein kinase superfamily protein [*Zea mays*]	*Sspon.04G0001770-2B*

## Data Availability

The original contributions presented in this study are included in the article/Supplementary Materials. Further inquiries can be directed to the corresponding authors.

## Supplementary Materials

The following supporting information can be downloaded at https://www.mdpi.com/article/10.3390/plants15193000/s1, Figure S1: Histogram of the broad-sense heritability (h_B_^2^) of the four leaf morphology traits. Four environments were H1 (Wengyuan, June–July 2023), H2 (Wengyuan, September 2023), H3 (Zhanjiang, September 2023), and H4 (Wengyuan, July–August 2022). Four leaf morphology traits were leaf length (LL), leaf width (LW), leaf length-to-width ratio (LR), and leaf area (LA). Figure S2: Histogram of the broad-sense heritability (h_B_^2^) of the five chlorophyll-related traits. Four environments were H1 (Wengyuan, June–July 2023), H2 (Wengyuan, September 2023), H3 (Zhanjiang, September 2023), and H4 (Wengyuan, July–August 2022). Five chlorophyll-related traits were chlorophyll a content (Cha), chlorophyll b content (Chb), the ratio of chlorophyll a to chlorophyll b (ChR), total chlorophyll content (Chab), and SPAD value (SPV). Cha, Chb, ChR and Chab were not measured in H4 because analytical acetone required for pigment extraction was temporarily unavailable under tightened precursor-chemical procurement rules; only the non-destructive SPAD value (SPV) was recorded in that environment. Figure S3: Population structure of the sugarcane association panel inferred using Admixture. Panels show (top to bottom) seven to eleven subgroups across different K-values (Supplementary Note). Figure S4: Three-dimensional scatter plot of the first three principal components derived from principal component analysis (Supplementary Note). Figure S5: Kinship matrix heatmap illustrating genetic relatedness among the 219 sugarcane accessions (Supplementary Note). Figure S6: Manhattan plots of GWAS results for leaf width (LW) across four environments. (A) H1 (Wengyuan, June–July 2023); (B) H2 (Wengyuan, September 2023); (C) H3 (Zhanjiang, September 2023); (D) H4 (Wengyuan, July–August 2022). Each point represents a SNP; the y-axis shows −log_10_(*p*-value) from GEMMA linear mixed model (LMM). The blue dashed line indicates the genome-wide significance threshold (*p* = 1.5 × 10^−6^). Chromosome numbers are shown on the x-axis. Figure S7: Manhattan plots of GWAS results for leaf length-to-width ratio (LR) across four environments. (A) H1 (Wengyuan, June–July 2023); (B) H2 (Wengyuan, September 2023); (C) H3 (Zhanjiang, September 2023); (D) H4 (Wengyuan, July–August 2022). Each point represents a SNP; the y-axis shows −log_10_(*p*-value) from GEMMA linear mixed model (LMM). The blue dashed line indicates the genome-wide significance threshold (*p* = 1.5 × 10^−6^). Chromosome numbers are shown on the x-axis. Figure S8: Manhattan plots of GWAS results for leaf area (LA) across four environments. (A) H1 (Wengyuan, June–July 2023); (B) H2 (Wengyuan, September 2023); (C) H3 (Zhanjiang, September 2023); (D) H4 (Wengyuan, July–August 2022). Each point represents a SNP; the y-axis shows −log_10_(*p*-value) from GEMMA linear mixed model (LMM). The blue dashed line indicates the genome-wide significance threshold (*p* = 1.5 × 10^−6^). Chromosome numbers are shown on the x-axis. Figure S9: Quantile–quantile (Q–Q) plots of GWAS results for the four leaf morphology traits. Four environments were H1 (Wengyuan, June–July 2023), H2 (Wengyuan, September 2023), H3 (Zhanjiang, September 2023), and H4 (Wengyuan, July–August 2022). Panels show (left to right, top to bottom): leaf length (LL, cm), leaf width (LW, cm), leaf ratio LW/LL (LR, dimensionless), and leaf area (LA, cm^2^). Figure S10: Manhattan (left) and Quantile–quantile (Q–Q, right) plots of GWAS results for chlorophyll a content (Cha). H1 (Wengyuan, June–July 2023), H2 (Wengyuan, September 2023), H3 (Zhanjiang, September 2023). In left, each point represents a SNP; the y-axis shows −log_10_(*p*-value) from GEMMA linear mixed model (LMM). The blue dashed line indicates the genome-wide significance threshold (*p* = 1.5 × 10^−6^). Chromosome numbers are shown on the circle. Figure S11: Manhattan (left) and Quantile–quantile (Q–Q, right) plots of GWAS results for chlorophyll b content (Chb). H1 (Wengyuan, June–July 2023), H2 (Wengyuan, September 2023), H3 (Zhanjiang, September 2023). In left, each point represents a SNP; the y-axis shows −log_10_(*p*-value) from GEMMA linear mixed model (LMM). The blue dashed line indicates the genome-wide significance threshold (*p* = 1.5 × 10^−6^). Chromosome numbers are shown on the circle. Figure S12: Manhattan (left) and Quantile–quantile (Q–Q, right) plots of GWAS results for the ratio of chlorophyll a to chlorophyll b (ChR). H1 (Wengyuan, June–July 2023), H2 (Wengyuan, September 2023), H3 (Zhanjiang, September 2023). In left, each point represents a SNP; the y-axis shows −log_10_(*p*-value) from GEMMA linear mixed model (LMM). The blue dashed line indicates the genome-wide significance threshold (*p* = 1.5 × 10^−6^). Chromosome numbers are shown on the circle. Figure S13: Manhattan (left) and Quantile–quantile (Q–Q, right) plots of GWAS results for total chlorophyll content (Chab). H1 (Wengyuan, June–July 2023), H2 (Wengyuan, September 2023), H3 (Zhanjiang, September 2023). In left, each point represents a SNP; the y-axis shows −log_10_(*p*-value) from GEMMA linear mixed model (LMM). The blue dashed line indicates the genome-wide significance threshold (*p* = 1.5 × 10^−6^). Chromosome numbers are shown on the circle. Figure S14: Manhattan (left) and Quantile–quantile (Q–Q, right) plots of GWAS results for SPAD value (SPV). H1 (Wengyuan, June–July 2023), H2 (Wengyuan, September 2023), H3 (Zhanjiang, September 2023), H4 (Wengyuan, July–August 2022). In left, each point represents a SNP; the y-axis shows −log_10_(*p*-value) from GEMMA linear mixed model (LMM). The blue dashed line indicates the genome-wide significance threshold (*p* = 1.5 × 10^−6^). Chromosome numbers are shown on the circle. Table S1: Phenotypic data of the sugarcane association panel comprising 219 accessions evaluated across four environments. Table S2: Variation in nine photosynthesis-related traits within the sugarcane association panel of 219 accessions across four environments. Table S3: GWAS-associated loci (intervals) identified by GWAS for nine photosynthesis-related traits in the sugarcane association panel. Table S4: Predicted candidate genes associated with photosynthesis-related traits and their functional annotations. Table S5: Simple correlation coefficients between the gas exchange parameters and the leaf morphology/chlorophyll traits across all tested environments. Table S6: Information on the sugarcane association panel of 219 clones. Table S7: Genomic inflation factors (λ) for all trait–environment combinations. Table S8: Ten most-prioritized candidates with their CDS, which are potentially associated with leaf photosynthesis.

## Abbreviations

GWAS	Genome-wide association studies
SNP	Single nucleotide polymorphism
PPR	Pentatricopeptide repeat-containing
MGDG	Monogalactosyldiacylglycerol
OR	Orange
PPD	PsbP domain-containing
CSP41b	Chloroplast stem-loop binding protein of 41 kDa b
QTL	Quantitative trait locus
G×E	Genotype × environment
LL	Leaf length
LW	Leaf width
LR	Leaf length-to-width ratio
LA	Leaf area
Cha	Chlorophyll a content
Chb	Chlorophyll b content
ChR	The ratio of chlorophyll a to chlorophyll b
Chab	Total chlorophyll content
SPV	SPAD value
λ	Genomic inflation factor
ANOVA	Analysis of Variance
hB2	Broad-sense heritability
LMM	Linear mixed model
Q-Q	Quantile–quantile
LSD	Least significant difference
MAF	Minor allele frequency
PCA	Principal component analysis
PVE	Phenotypic variance explained
LD	Linkage disequilibrium
R4	The forth ratoon
R3	The third ratoon
