# Supplementary material for: GWAS of Leaf Morphological and Chlorophyll-Related Traits Provides Genetic Resources for Improving Photosynthetic Traits in Sugarcane
Source: Plants (Basel). 2026 Sep 30;15(19):3000. doi: 10.3390/plants15193000 (PMC13667283; doi:10.3390/plants15193000)
Supplement: Supplementary file 1 [file plants-15-03000-s001.zip › Chen_Supplementary_Figure_Note_Text_Revised_20260914.pdf]

Supplementary information

GWAS of leaf morphological and chlorophyll-related traits provides genetic resources for improving photosynthetic traits in sugarcane

Contents

Figure S1. Histogram of the broad-sense heritability (h_B^2) of the four leaf morphology traits........3
Figure S2. Histogram of the broad-sense heritability (h_B^2) of the five chlorophyll-related traits. ...4
Figure S3. Population structure of the sugarcane association panel inferred using Admixture. 5
Figure S4. Three-dimensional scatter plot of the first three principal components derived from principal component analysis (Supplementary Note). 6
Figure S5. Kinship matrix heatmap illustrating genetic relatedness among the 219 sugarcane accessions (Supplementary Note). 7
Figure S6. Manhattan plots of GWAS results for leaf width (LW) across four environments. 8
Figure S7. Manhattan plots of GWAS results for leaf length-to-width ratio (LR) across four environments...9
Figure S8. Manhattan plots of GWAS results for leaf area (LA) across four environments..........10
Figure S9. Quantile − quantile (Q − Q) plots of GWAS results for the four leaf morphology traits. 11
Figure S10. Manhattan (left) and Quantile − quantile (Q − Q, right) plots of GWAS results for chlorophyll a content (Cha)...12
Figure S11. Manhattan (left) and Quantile − quantile (Q − Q, right) plots of GWAS results for chlorophyll b content (Chb). 12
Figure S12. Manhattan (left) and Quantile − quantile (Q − Q, right) plots of GWAS results for the ratio of chlorophyll a to chlorophyll b (ChR). 13
Figure S13. Manhattan (left) and Quantile − quantile (Q − Q, right) plots of GWAS results for total chlorophyll content (Chab). 13
Figure S14. Manhattan (left) and Quantile − quantile (Q − Q, right) plots of GWAS results for SPAD value (SPV)...14
Supplementary Note. Population structure, principal component, and kinship analysis 14
Supplementary Text 14

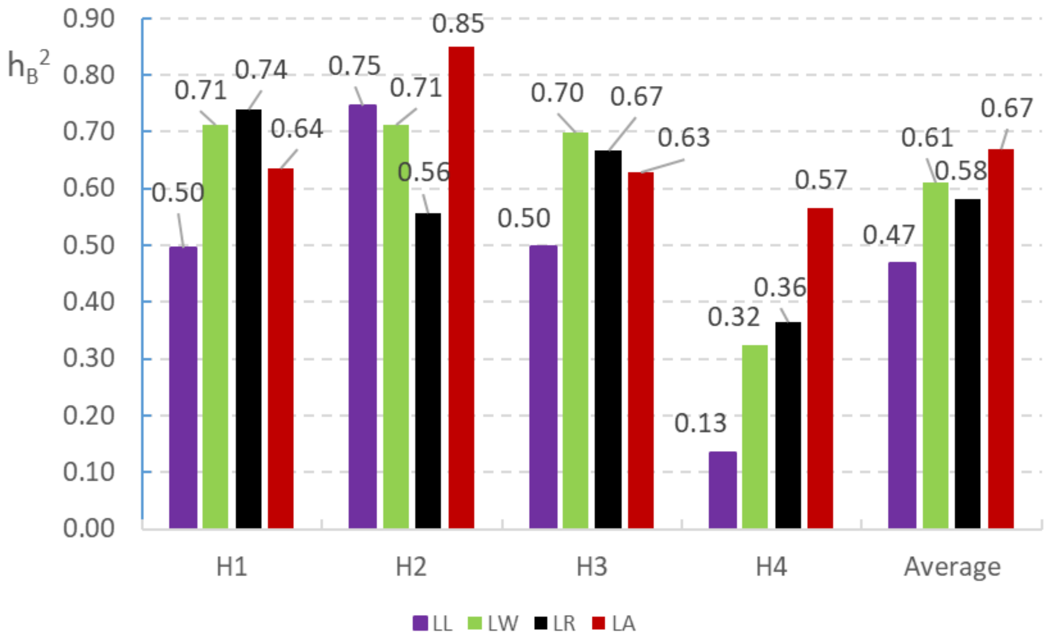

Figure S1. Histogram of the broad-sense heritability (h_B^2) of the four leaf morphology traits.

Four environments were H1 (Wengyuan, June-July 2023), H2 (Wengyuan, September 2023), H3 (Zhanjiang, September 2023), and H4 (Wengyuan, July-August 2022). Four leaf morphology traits were leaf length (LL), leaf width (LW), leaf length-to-width ratio (LR), and leaf area (LA).

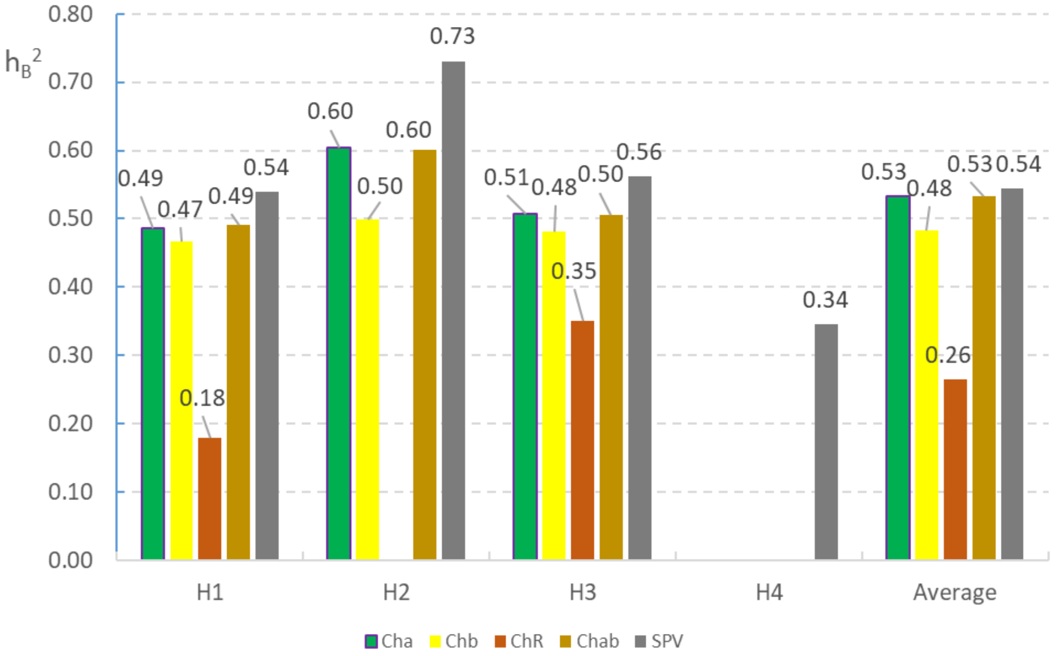

Figure S2. Histogram of the broad-sense heritability (h_B^2) of the five chlorophyll-related traits.

Four environments were H1 (Wengyuan, June-July 2023), H2 (Wengyuan, September 2023), H3 (Zhanjiang, September 2023), and H4 (Wengyuan, July-August 2022). Five chlorophyll-related traits were chlorophyll a content (Cha), chlorophyll b content (Chb), the ratio of chlorophyll a to chlorophyll b (ChR), total chlorophyll content (Chab), and SPAD value (SPV). Cha, Chb, ChR and Chab were not measured in H4 because analytical acetone required for pigment extraction was temporarily unavailable under tightened precursor-chemical procurement rules; only the non-destructive SPAD value (SPV) was recorded in that environment.

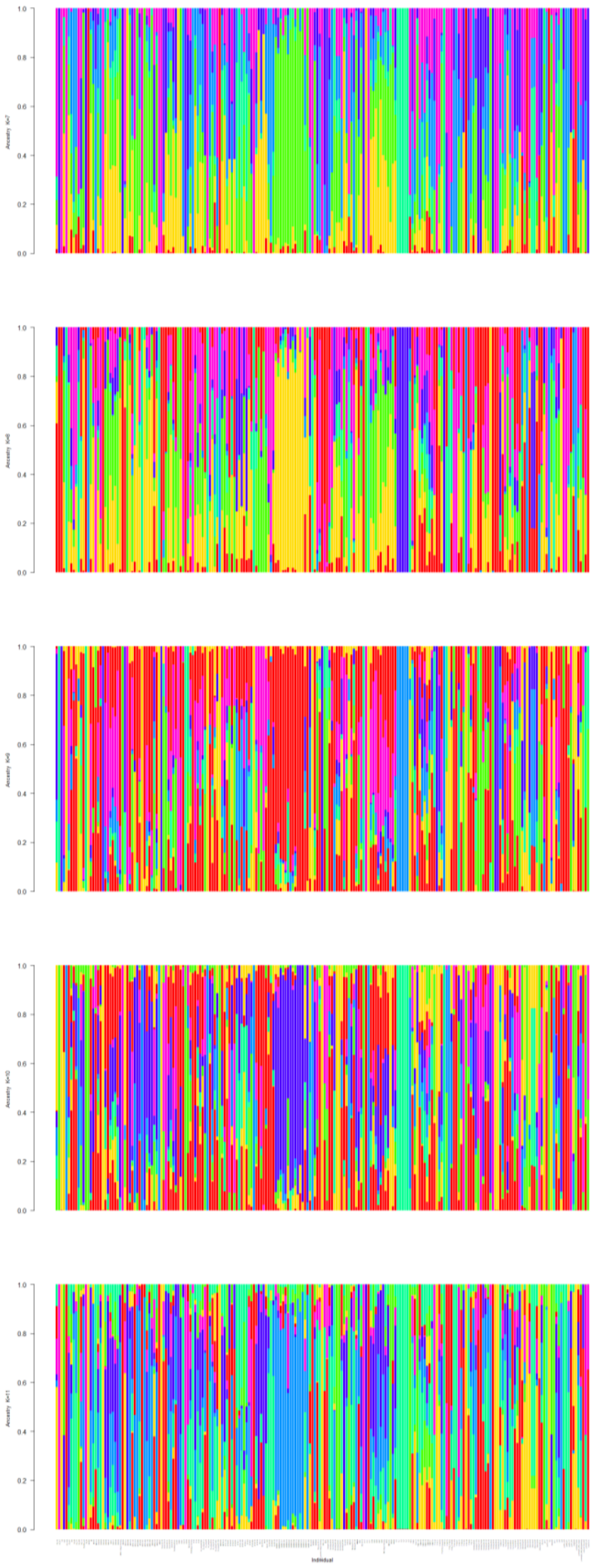

Figure S3. Population structure of the sugarcane association panel inferred using Admixture.

Panels show (top to bottom) seven to eleven subgroups across different K-values (Supplementary Note)

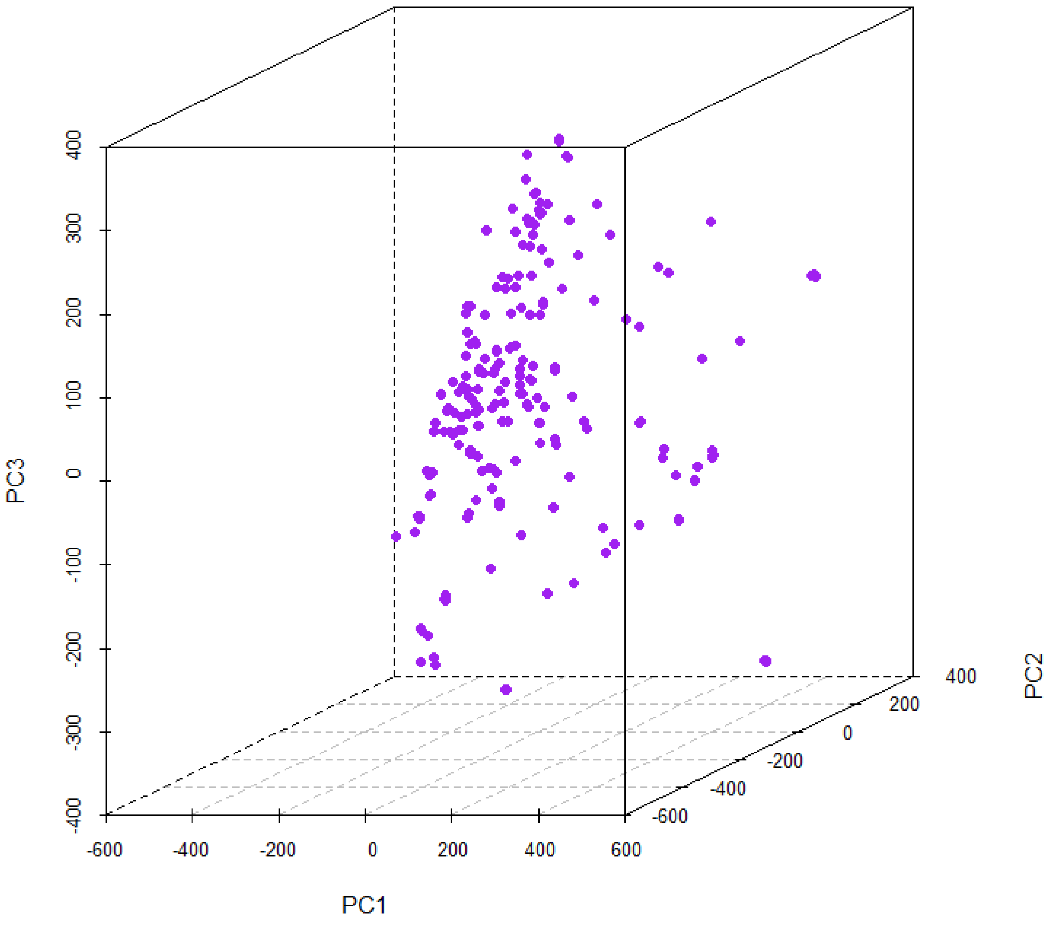

Figure S4. Three-dimensional scatter plot of the first three principal components derived from principal component analysis (Supplementary Note).

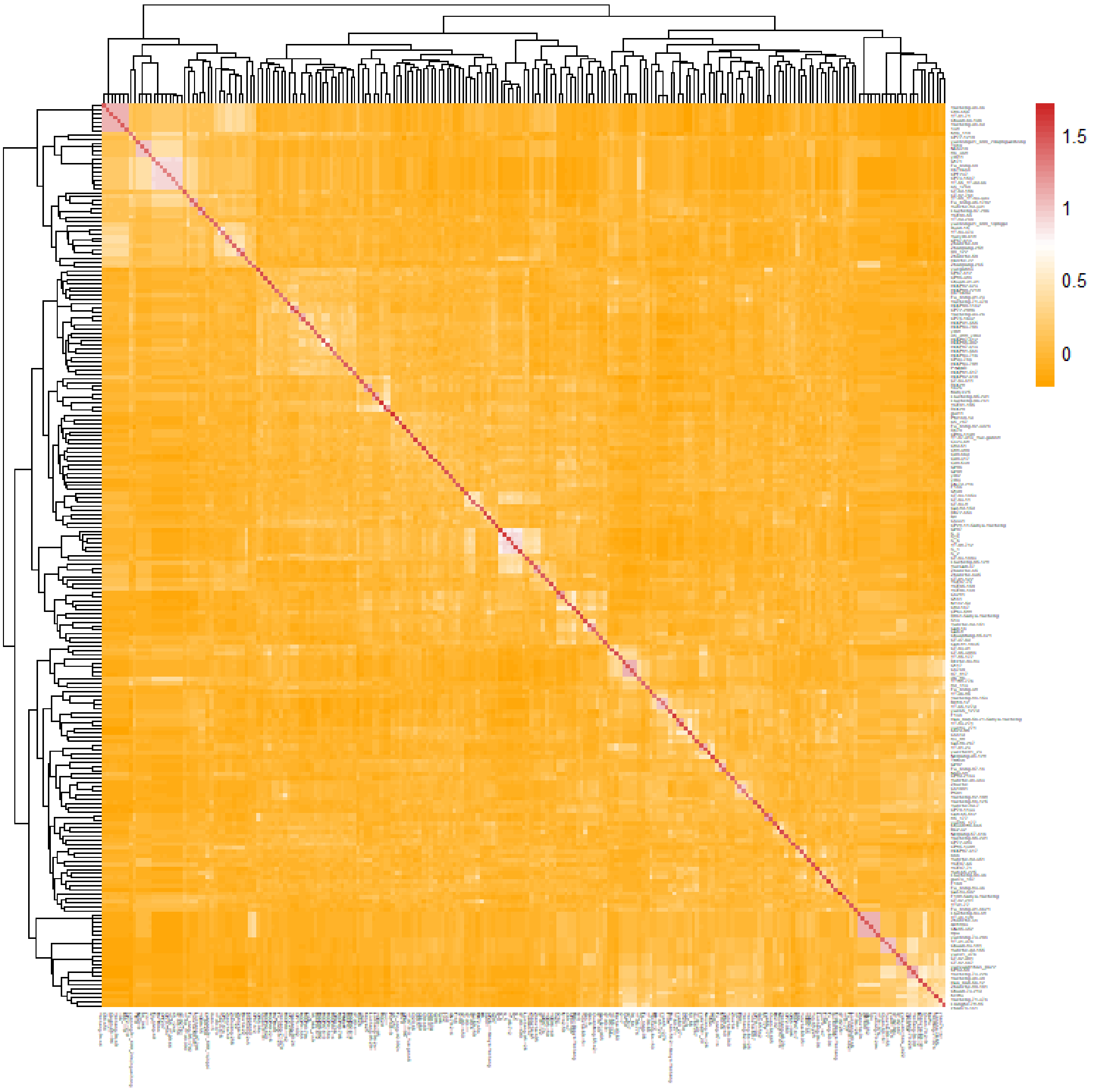

Figure S5. Kinship matrix heatmap illustrating genetic relatedness among the 219 sugarcane accessions (Supplementary Note).

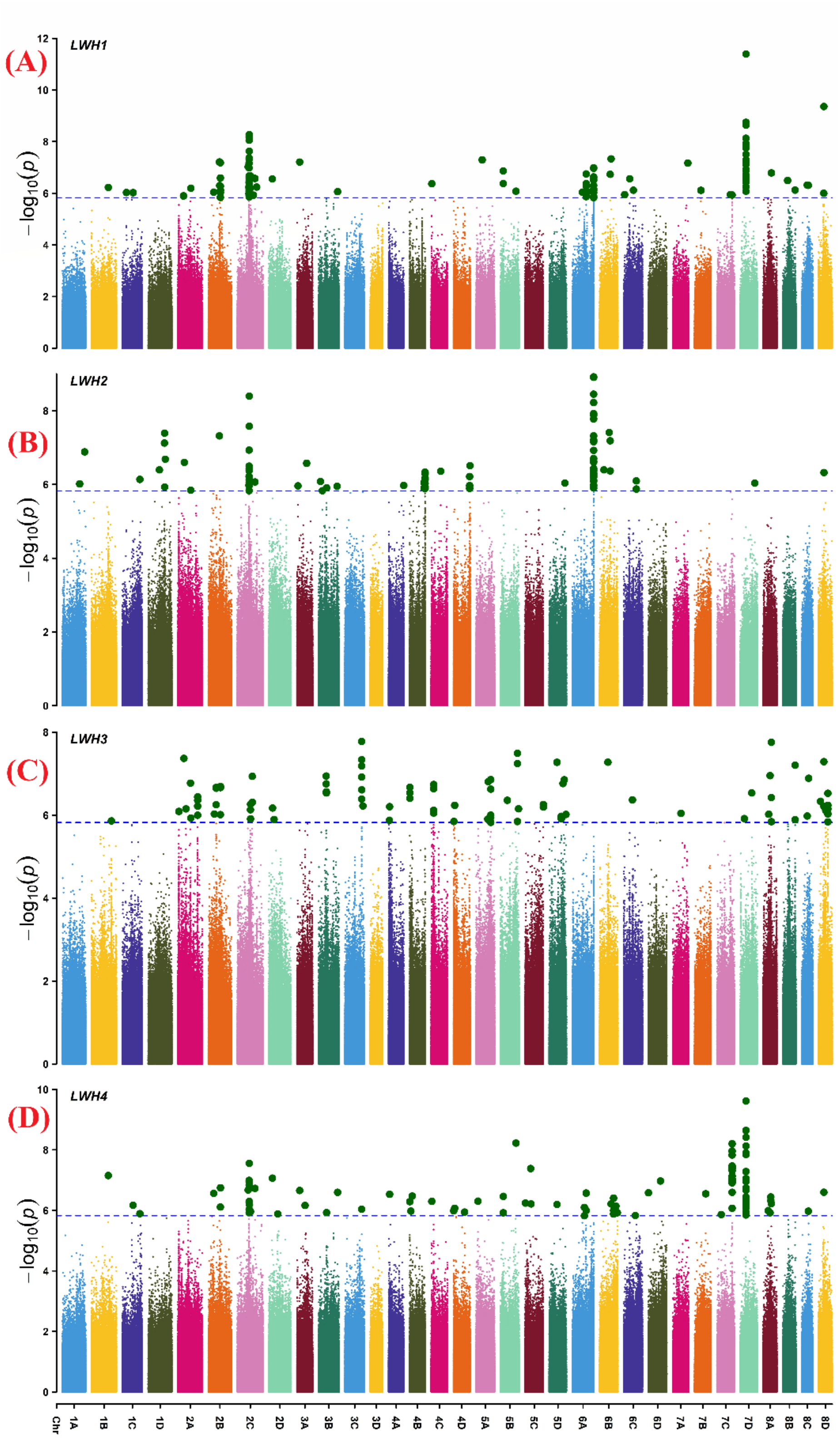

Figure S6. Manhattan plots of GWAS results for leaf width (LW) across four environments.

(A) H1 (Wengyuan, June-July 2023); (B) H2 (Wengyuan, September 2023); (C) H3 (Zhanjiang, September 2023); (D) H4 (Wengyuan, July-August 2022). Each point represents a SNP; the y-axis shows $-\log_{10}$(*P*-value) from GEMMA linear mixed model (LMM). The blue dashed line indicates the genome-wide significance threshold ($P = 1.5 \times 10^{-6}$). Chromosome numbers are shown on the x-axis.

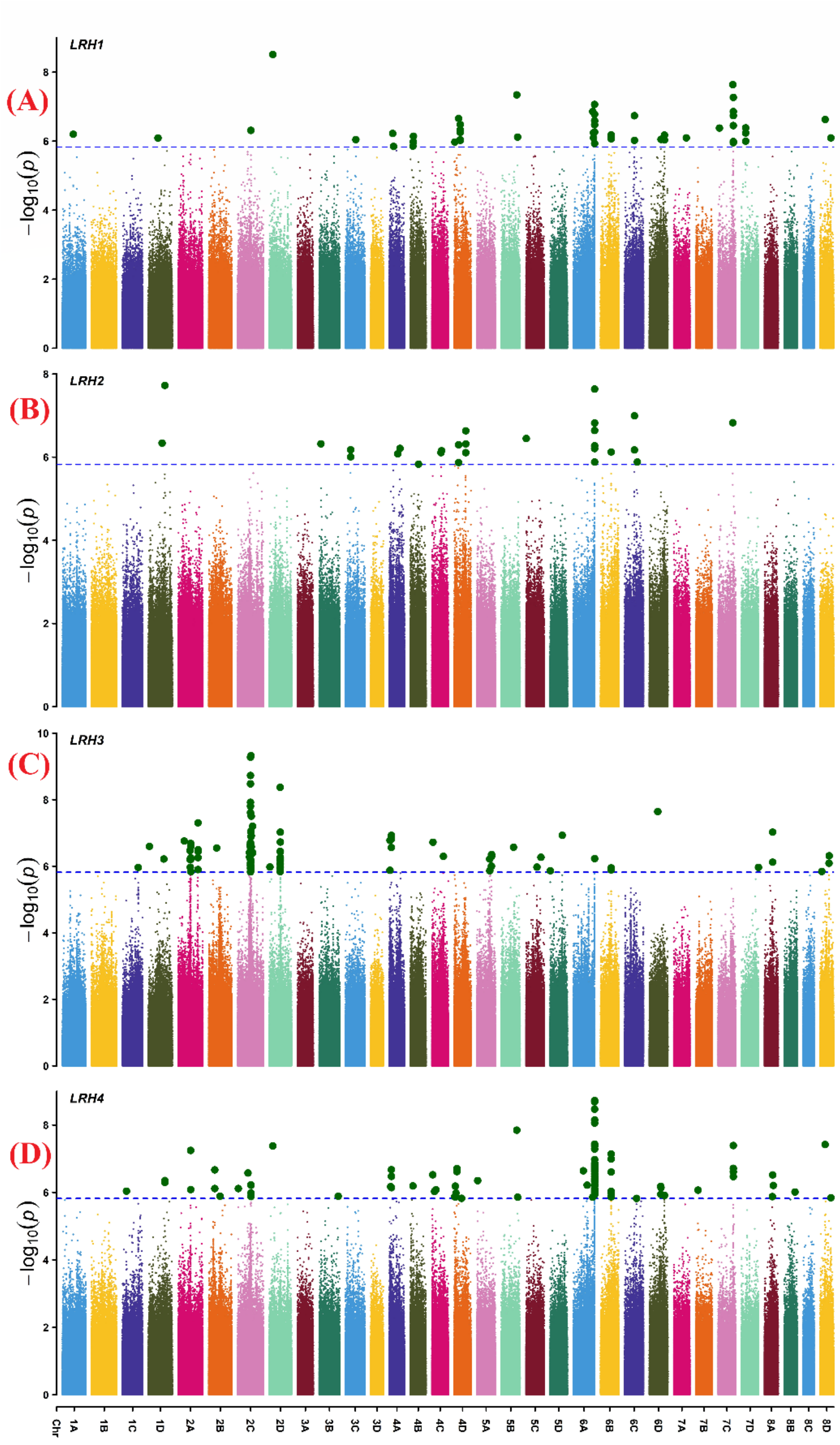

Figure S7. Manhattan plots of GWAS results for leaf length-to-width ratio (LR) across four environments.

(A) H1 (Wengyuan, June-July 2023); (B) H2 (Wengyuan, September 2023); (C) H3 (Zhanjiang, September 2023); (D) H4 (Wengyuan, July-August 2022). Each point represents a SNP; the y-axis shows $-\log_{10}$(*P*-value) from GEMMA linear mixed model (LMM). The blue dashed line indicates the genome-wide significance threshold ($P = 1.5 \times 10^{-6}$). Chromosome numbers are shown on the x-axis.

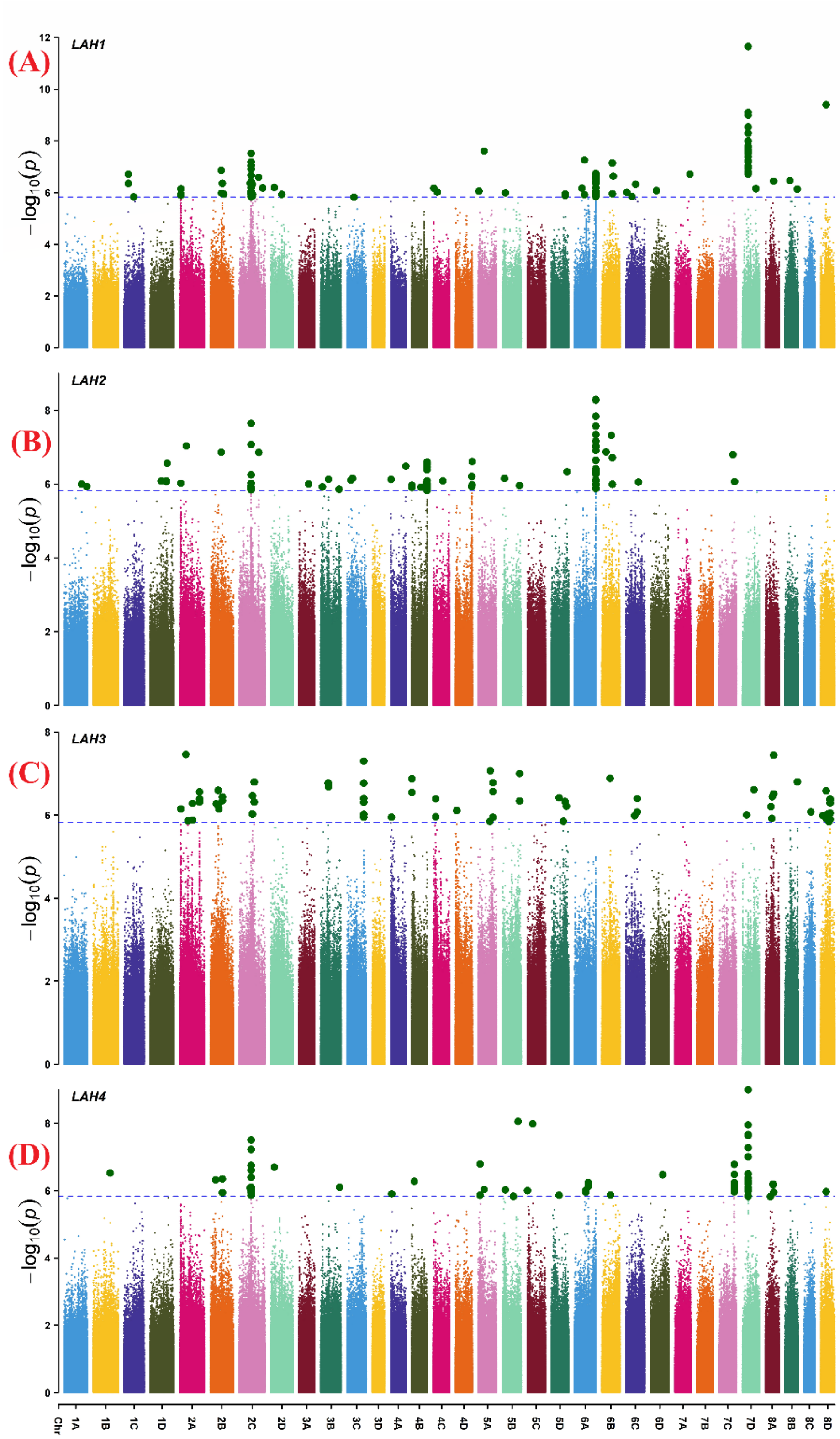

Figure S8. Manhattan plots of GWAS results for leaf area (LA) across four environments.

(A) H1 (Wengyuan, June-July 2023); (B) H2 (Wengyuan, September 2023); (C) H3 (Zhanjiang, September 2023); (D) H4 (Wengyuan, July-August 2022). Each point represents a SNP; the y-axis shows -$\log_{10}$(P-value) from GEMMA linear mixed model (LMM). The blue dashed line indicates the genome-wide significance threshold (P = 1.5×10^{-6}). Chromosome numbers are shown on the x-axis.

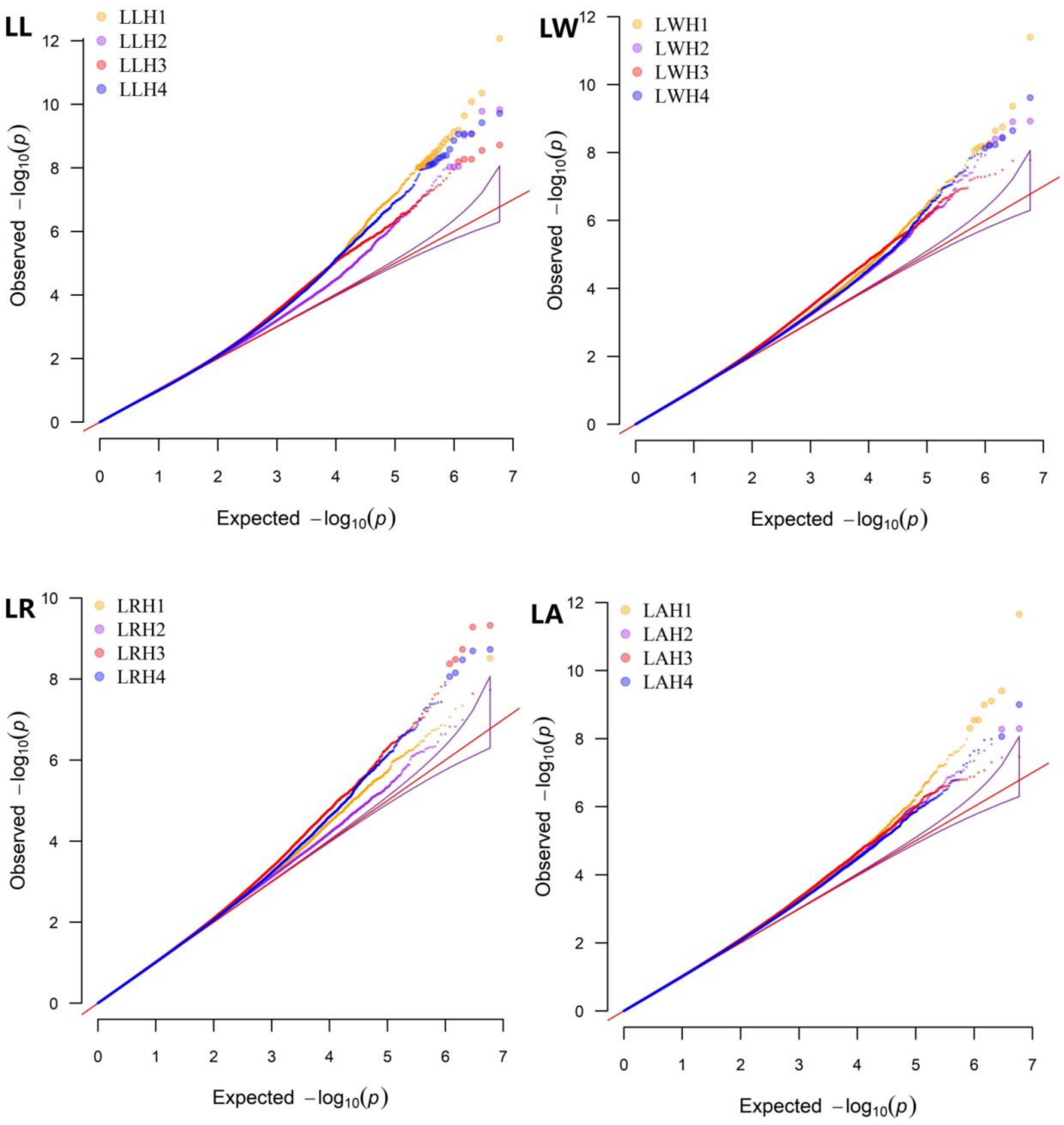

Figure S9. Quantile–quantile (Q–Q) plots of GWAS results for the four leaf morphology traits.

Four environments were H1 (Wengyuan, June-July 2023), H2 (Wengyuan, September 2023), H3 (Zhanjiang, September 2023), and H4 (Wengyuan, July-August 2022). Panels show (left to right, top to bottom): leaf length (LL, cm), leaf width (LW, cm), leaf ratio LW/LL (LR, dimensionless), and leaf area (LA, cm^2).

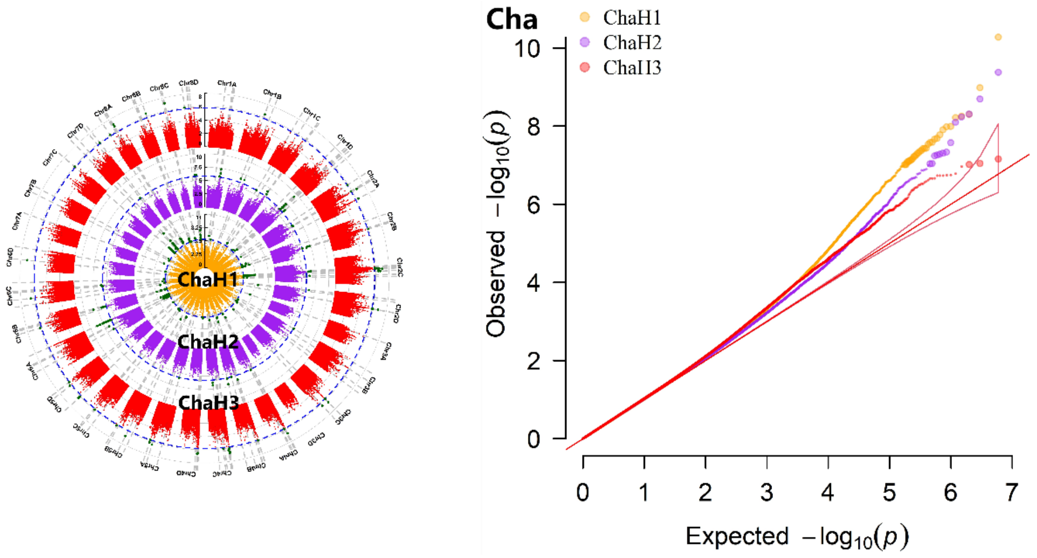

Figure S10. Manhattan (left) and Quantile–quantile (Q–Q, right) plots of GWAS results for chlorophyll a content (Cha).

H1 (Wengyuan, June-July 2023), H2 (Wengyuan, September 2023), H3 (Zhanjiang, September 2023). In left, each point represents a SNP; the y-axis shows -$\log_{10}$(P-value) from GEMMA linear mixed model (LMM). The blue dashed line indicates the genome-wide significance threshold (P = 1.5 × 10^{-6}). Chromosome numbers are shown on the circle.

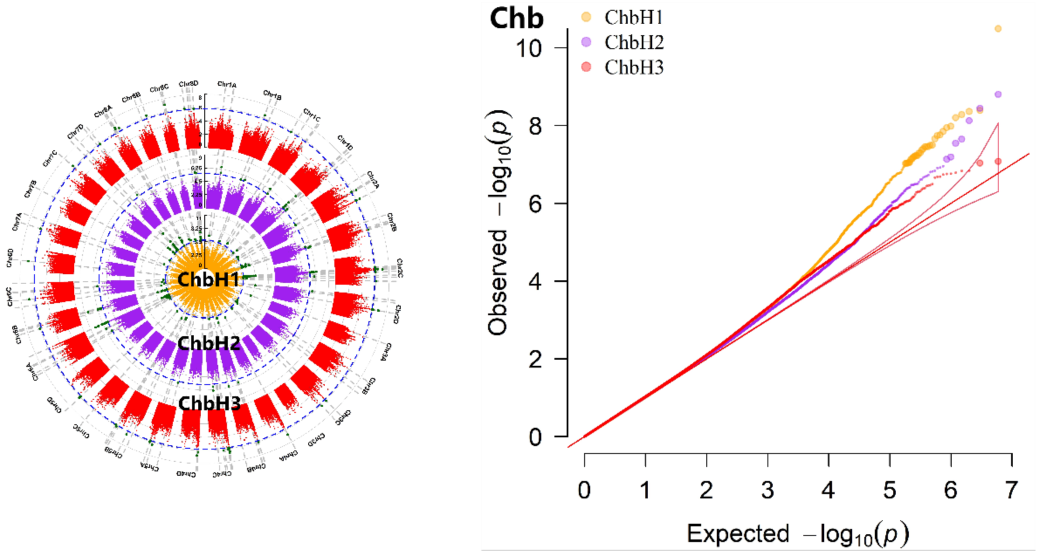

Figure S11. Manhattan (left) and Quantile–quantile (Q–Q, right) plots of GWAS results for chlorophyll b content (Chb).

H1 (Wengyuan, June-July 2023), H2 (Wengyuan, September 2023), H3 (Zhanjiang, September 2023). In left, each point represents a SNP; the y-axis shows -$\log_{10}$(P-value) from GEMMA linear mixed model (LMM). The blue dashed line indicates the genome-wide significance threshold (P = 1.5 × 10^{-6}). Chromosome numbers are shown on the circle.

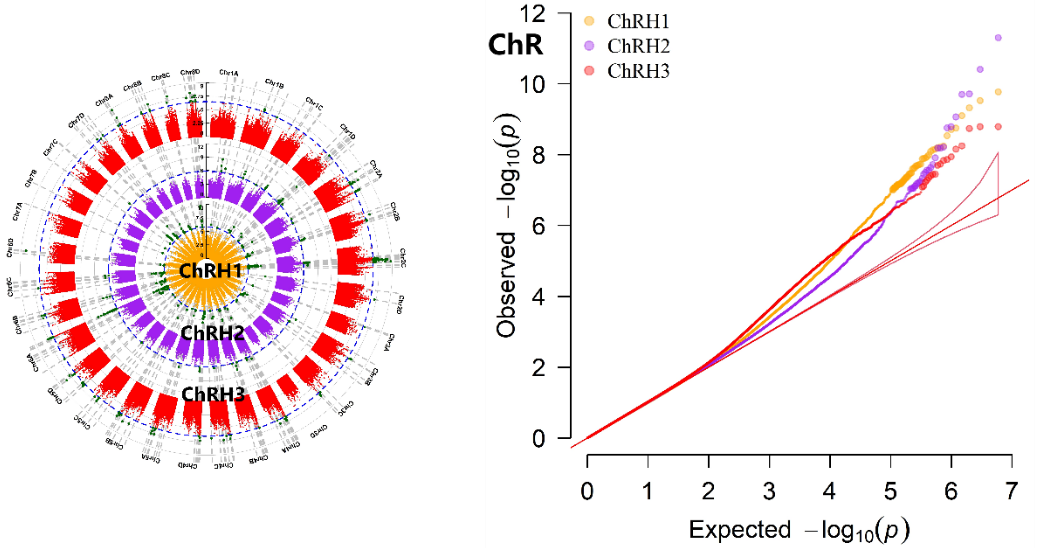

Figure S12. Manhattan (left) and Quantile–quantile (Q–Q, right) plots of GWAS results for the ratio of chlorophyll a to chlorophyll b (ChR).

H1 (Wengyuan, June-July 2023), H2 (Wengyuan, September 2023), H3 (Zhanjiang, September 2023). In left, each point represents a SNP; the y-axis shows -log$_{10}$(P-value) from GEMMA linear mixed model (LMM). The blue dashed line indicates the genome-wide significance threshold (P = 1.5 × 10^{-6}). Chromosome numbers are shown on the circle.

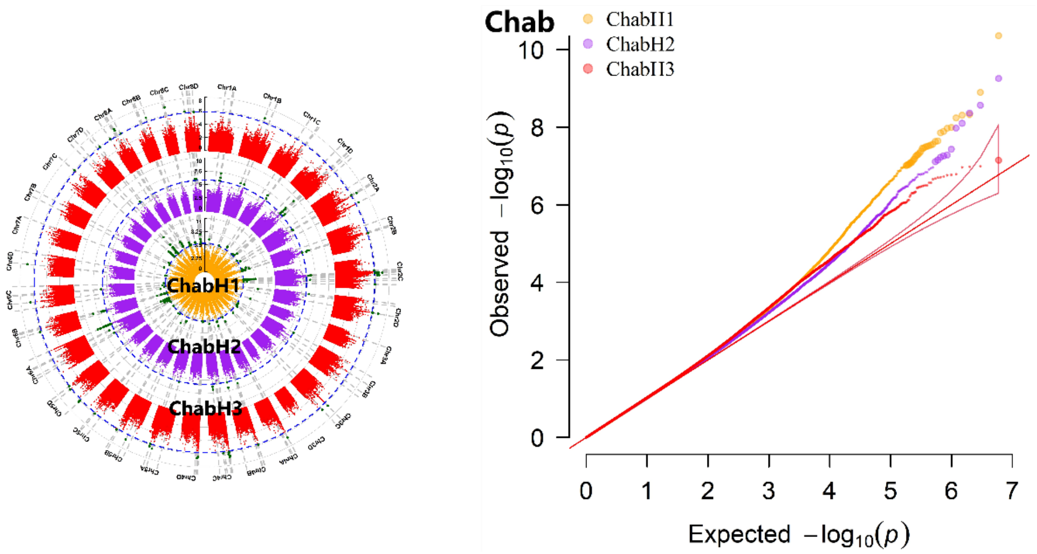

Figure S13. Manhattan (left) and Quantile–quantile (Q–Q, right) plots of GWAS results for total chlorophyll content (Chab).

H1 (Wengyuan, June-July 2023), H2 (Wengyuan, September 2023), H3 (Zhanjiang, September 2023). In left, each point represents a SNP; the y-axis shows -log10 (P-value) from GEMMA linear mixed model (LMM). The blue dashed line indicates the genome-wide significance threshold (P = 1.5 × 10^{-6}). Chromosome numbers are shown on the circle.

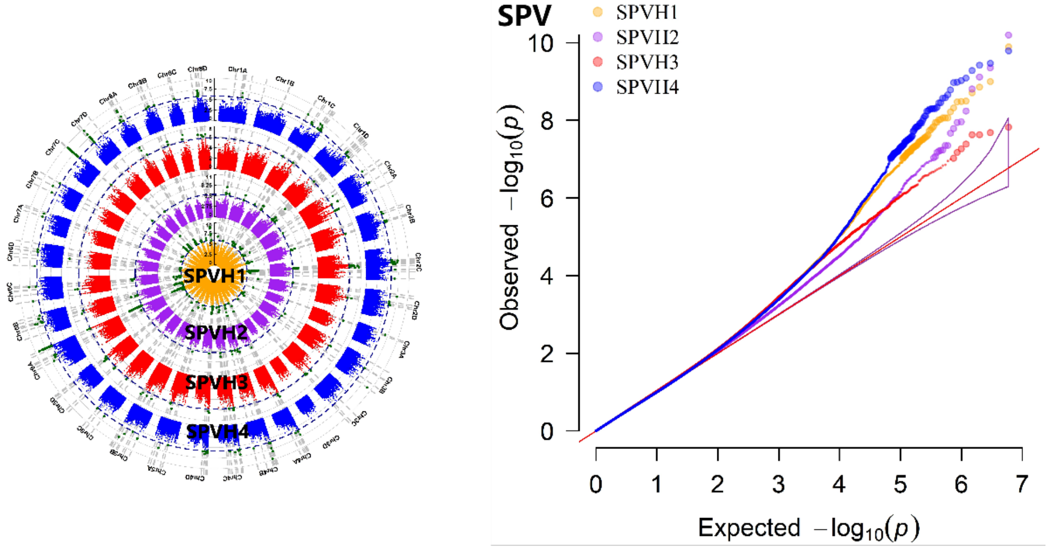

Figure S14. Manhattan (left) and Quantile–quantile (Q–Q, right) plots of GWAS results for SPAD value (SPV).

H1 (Wengyuan, June-July 2023), H2 (Wengyuan, September 2023), H3 (Zhanjiang, September 2023), H4 (Wengyuan, July-August 2022). In left, each point represents a SNP; the y-axis shows $-\log_{10}$(*P*-value) from GEMMA linear mixed model (LMM). The blue dashed line indicates the genome-wide significance threshold ($P = 1.5 \times 10^{-6}$). Chromosome numbers are shown on the circle.

Supplementary Note. Population structure, principal component, and kinship analysis

Population structure was inferred using ADMIXTURE software (v1.3.0; http://software.genetics.ucla.edu/admixture/). Principal component analysis (PCA) and estimation of the kinship matrix (K matrix) were performed in TASSEL 5.0 based on the complete set of high-quality SNPs.

Supplementary Text

For the four leaf morphology traits, LL ranged from 93.67 to 163.00 cm in H1, 104.50 to 184.00 cm in H2, 118.67 to 183.17 cm in H3, and 92.51 to 143.02 cm in H4, with mean values of 132.97, 148.98, 150.37, and 118.31 cm, respectively. LW varied from 2.66 to 6.19 cm (H1), 2.58 to 6.88

cm (H2), 2.91 to 6.36 cm (H3), and 2.36 to 8.18 cm (H4), with corresponding means of 4.33, 4.61, 4.62, and 3.77 cm. LR ranged from 20.88 to 48.40 (H1), 20.40 to 52.10 (H2), 23.44 to 50.14 (H3), and 19.98 to 52.64 (H4), with mean values of 31.66, 33.39, 33.54, and 32.42, respectively. LA ranged from 140.41 to 455.81 cm^2 (H1), 140.59 to 536.59 cm^2 (H2), 193.30 to 494.28 cm^2 (H3), and 121.98 to 513.71 cm^2 (H4), with mean values of 307.04, 318.87, 342.25, and 220.90 cm^2, respectively (Table S2 and Figure 3).

For the five chlorophyll-related traits, Cha ranged from 2.99 to 6.21 $mg \cdot dm^{-2}$ (H1), 2.75 to 6.90 $mg \cdot dm^{-2}$ (H2), and 3.28 to 6.40 $mg \cdot dm^{-2}$ (H3), with mean values of 4.63, 4.43, and 4.65 $mg \cdot dm^{-2}$, respectively. Chb ranged from 0.92 to 2.14 $mg \cdot dm^{-2}$ (H1), 0.92 to 2.50 $mg \cdot dm^{-2}$ (H2), and 0.98 to 2.05 $mg \cdot dm^{-2}$ (H3), with mean values of 1.47, 1.46, and 1.48 $mg \cdot dm^{-2}$. ChR ranged from 2.76 to 3.61 (H1), 2.72 to 3.35 (H2), and 2.78 to 3.88 (H3), with corresponding means of 3.17, 3.04, and 3.16. Chab ranged from 4.01 to 8.29 $mg \cdot dm^{-2}$ (H1), 3.68 to 9.40 $mg \cdot dm^{-2}$ (H2), and 4.30 to 8.36 $mg \cdot dm^{-2}$ (H3), with mean values of 6.10, 5.89, and 6.13 $mg \cdot dm^{-2}$. SPV ranged from 36.79 to 52.38 (H1), 31.45 to 53.05 (H2), 33.66 to 51.17 (H3), and 33.13 to 52.07 (H4), with mean values of 44.28, 42.32, 43.13, and 42.84, respectively (Table S2 and Figure 3).
